# Identification of factors involved in dimorphism and pathogenicity of *Zymoseptoria tritici*

**DOI:** 10.1371/journal.pone.0183065

**Published:** 2017-08-22

**Authors:** Alexander Yemelin, Annamaria Brauchler, Stefan Jacob, Julian Laufer, Larissa Heck, Andrew J. Foster, Luis Antelo, Karsten Andresen, Eckhard Thines

**Affiliations:** 1 Institute for Biotechnology and Drug Research (IBWF gGmbH), Kaiserslautern, Germany; 2 Institute of Molecular Physiology, Microbiology and Wine Research, Johannes Gutenberg University Mainz, Mainz, Germany; University of Nebraska-Lincoln, UNITED STATES

## Abstract

A forward genetics approach was applied in order to investigate the molecular basis of morphological transition in the wheat pathogenic fungus *Zymoseptoria tritici*. *Z*. *tritici* is a dimorphic plant pathogen displaying environmentally regulated morphogenetic transition between yeast-like and hyphal growth. Considering the infection mode of *Z*. *tritici*, the switching to hyphal growth is essential for pathogenicity allowing the fungus the host invasion through natural openings like stomata. We exploited a previously developed *Agrobacterium tumefaciens*-mediated transformation (ATMT) to generate a mutant library by insertional mutagenesis including more than 10,000 random mutants. To identify genes involved in dimorphic switch, a plate-based screening system was established. With this approach eleven dimorphic switch deficient random mutants were recovered, ten of which exhibited a yeast-like mode of growth and one mutant predominantly growing filamentously, producing high amount of mycelium under different incubation conditions. Using genome walking approach previously established, the T-DNA integration sites were recovered and the disrupted genomic loci of corresponding mutants were identified and validated within reverse genetics approach. As prove of concept, two of the random mutants obtained were selected for further investigation using targeted gene inactivation. Both genes deduced were found to encode known factors, previously characterized in other fungi: Ssk1p being constituent of HOG pathway and Ade5,7p involved in *de novo* purine biosynthesis. The targeted mutant strains defective in these genes exhibit a drastically impaired virulence within infection assays on whole wheat plants. Moreover exploiting further physiological assays the predicted function for both gene products could be confirmed in concordance with conserved biological role of homologous proteins previously described in other fungal organisms.

## Introduction

*Zymoseptoria tritici* (teleomorph: *Mycosphaerella graminicola*, syn. *Septoria tritici*) is ascomycete that causes the most devastating foliar disease of bread and durum wheat in temperate climates worldwide, known as the *Septoria tritici* blotch (STB). Septoria wheat diseases have increased in incidence over the last few decades despite the deployment of fungicide treatments and *Z*. *tritici* is consistently the most destructive foliar pathogen of wheat in Europe [1–4]. STB has a substantial economic impact leading to annual yield losses of up to 50%, representing a significant threat to global food production. Solely in Europe annual losses from STB are estimated to $400 million dollars [5,6]. The increased prevalence of these diseases is considered to be due to the more frequent use of high yielding semi-dwarf rust-resistant cultivars, increased nitrogen application rates and changes to post harvest practices [7,8].

Although cultivars with improved resistance have been developed, their yield has to date not matched that of fungicide treated susceptible varieties and control of the disease by fungicide application [9–12]. At the present crop protection management in terms to control the STB generally relies on azole fungicides (e.g. methyl benzimidazole carbamates (MBCs), demethylase inhibitors (DMIs)) or the quinone outside inhibitors (QoIs) commonly referred as strobilurins [13–16]. However, the control of STB is complicated due to the extremely high levels of genetic variability of the *Z*. *tritici* populations enabling rapid adaptation and increased insensitivity to fungicides [13,17,18]. Given the trend towards emergence of resistant isolates, there is a clear need for the development of novel plant protective agents to combat *Septoria* leaf blotch. This requires, however, a full understanding of the molecular mechanisms governing the virulence-related processes of *Z*. *tritici* in order to develop new plant protection strategies.

Looking at the infection cycle of *Z*. *tritici* we can accentuate three main phases of development: entry of the fungus, colonization of the plant tissue and formation of fruiting bodies [19]. In contrast to e.g. the rice pathogen *Magnaporthe oryzae*, *Z*. *tritici* penetrates its host through the stomata and does not form appressoria. Therefore transition from a yeast-like to a filamentous form, also known as dimorphic switch, appears essential prerequisite for plant infection [8]. Moreover, the fungus exhibits an *in vitro* triggered yeast-to-hyphal transition, allowing investigation of this process at molecular level. It should be noticed, that ability of dimorphic switch is not exclusively restricted to *Z*. *triciti*, but also observed in a range of other fungal organisms, including systemic dimorphic fungi. However in contrast to other representatives of dimorphic fungi like *Ustilago maydis*, *Histoplasma capsulatum* and *Candida albicans*, the molecular mechanisms governing dimorphic transition in *Z*. *tritici* have not yet been addressed in greater detail. To date only a few genes having a role in dimorphism in *Z*. *tritici* have been functionally characterized [11,20]. Most of them represent components of conserved MAPK and cAMP-PKA pathways, regulating diverse physiological processes. Among them are *ZtHOG1*, encoding a well conserved mitogen-activated protein kinase ZtHog1p, which plays a crucial role in the osmotic stress response in many fungi [21], *ZtSLT2* and *ZtFUS3*, the homologs of which are essential for cell wall integrity and mating/pheromone response in *S*. *cerevisiae* respectively [22]. Mehrabi *et al*. have also previously demonstrated a role of conserved cAMP pathway in regulating dimorphism related processes. Thus disruption of two genes termed *ZtTpk2* and *ZtBcy1*, encoding catalytic and regulatory subunit respectively, influenced the switch ability of corresponding mutants. Further examples of factors having impact on dimorphism are heterotrimeric G-proteins and the intrinsic cAMP concentration. The three genes, encoding G-alpha Subunits (*ZtGpa1*, *ZtGpa2*, *ZtGpa3*), and one gene encoding Gβ (*ZtGpb1*) were previously functionally characterized. *ZtGpa1* was found to be essential for the negative regulation of mycelial growth, since the corresponding *ZtGpa1* null mutant attracted attention by the formation of much longer conidia on YEG- and PDA—solid media, which resulted in fluffy mycelia at later stadium. *ZtGpa3* deficient mutants exhibited a clearly pronounced filamentous growth. Both *ZtGpb1* and *ZtGpa3* deficient mutants had a reduced intracellular concentration of cAMP, indicating that products of both genes regulate cAMP concentration positively. The virulence of the corresponding mutant strains Δ*Ztgpa1*, Δ*Ztgpa3* and Δ*Ztgpb1* was significantly reduced in all cases [23]. Mutants lacking the corresponding genes were shown to grow yeast-like, being unable to infect the host plants. Together with the provided studies and the established role of *ZtHOG1*, *ZtFUS3* and *ZtSLT2* in dimorphism, these results lead to the conclusion, that the ability to perform a morphogenetic transition appears essential for the pathogenic development of the fungus.

Two additional factors implicated in vegetative filamentous growth maintenance and dimorphic switch were previously identified by reverse genetics studies in *Z*. *tritici*. Thus, inactivation of the cyclin-encoding gene *ZtMCC* (previously published as *MgMCC*), orthologous to *Fusarium verticillioides FCC1* is associated with a delayed filamentous growth, unusual hyphal swellings, increased melanin biosynthesis, stress tolerance and reduced pathogenicity [24]. Finally, exploiting a random mutagenesis approach, the gene *ZtALG2* (previously published as *MgALG2*) was identified and functionally characterized, indicating its involvement in glycosylation of secreted proteins. Deletion of *ZtALG2* led to a mutant unable to infect wheat. Furthermore, the mutant is impaired in switching from yeast to filamentous growth and protein secretion [25].

By addressing dimorphism in this study, we aimed to unravel genetic determinants and pathogenicity factors regulating dimorphic transition in the plant pathogenic fungus *Z*. *tritici*. Interruption of this critical differentiation process within the pathogenic development could contribute to modern and innovative plant protection strategies to combat the disease. In the present study, we report the application of ATMT for insertional mutagenesis of *Z*. *tritici* to generate more than 10,000 hygromycin resistant transformants, followed by screening for defects in the ability to perform the dimorphic transition. The previously described genome-walking method [26] aided the identification of disrupted gene loci associated with corresponding genes, hypothesized to be involved in the dimorphism associated process. The availability of the *Z*. *tritici* genome sequence and the specificity of genome walking approach to recover the T-DNA tags and the flanking genomic sequence facilitated the identification of the precise genomic T-DNA insertion position by BLAST interrogation against the local genome database of the wildtype strain IPO323. The sequence analysis of the disrupted genes led to the isolation of eleven different dimorphism-related genes, two of which were functionally characterized in this study.

## Material and methods

### Strains, growth conditions, and oligonucleotides

All mutants described in this study are derived from the Dutch field isolate *Zymoseptoria tritici* IPO323, used as a wildtype strain (CBS Fungal Collection, Utrecht, NL). IPO323 and the mutant strains were routinely cultivated at 18°C and 120 rpm in liquid medium YEG (Glucose 10 g/l, yeast extract 10 g/l, pH 6.5) or were grown at 18°C on YEG-agar medium (2%). As basis for phenotypical screening and stress based growth assays N-deprivation, MM, YEG and PDA agar media were used. For the stress assays, H_2_O_2_, SDS, Congo red, the phenylpyrrole fungicide fludioxonil, NaCl, and sorbitol were added to YEG medium at the concentrations indicated in the figure legends. The assays were carried out in triplicate and were repeated three times. Nitrogen starvation medium (N-deprivation) (pH 6.5), which represented the switch-inducing medium, contained 10 g/l glucose, 0.25 ml/l of a 0.01% biotin solution, 50 ml/l of a salt solution without nitrate salts (10.4 g/l KCl, 30.4 g/l KH_2_PO_4_, 10.4 g/l MgSO_4_·7H_2_O), 1 ml/l of a trace element solution (22 g/l ZnSO_4_·7H_2_O, 11 g/l H_3_BO_3_, 5 g/l MnCl_2_·4H_2_O, 5 g/l FeSO_4_·7H_2_O, 1.7 g/l CoCl_2_·6H_2_O, 1.6 g/l CuSO_4_·5H_2_O, 1.5 g/l Na_2_MoO_4_·2H_2_O, 50 g/l Na_4_·EDTA) and 1 ml/l of a 1% thiamine dichloride solution. Minimal medium (MM) was prepared based on nitrogen starvation medium and additionally contained a nitrogen source in the form of nitrate salt solution (10.4 g/l KCl, 30.4 g/l KH_2_PO_4_, 10.4 g/l MgSO_4_·7H_2_O and 120 g/l NaNO_3_). Potato dextrose agar (PDA) was obtained from Carl Roth (Karlsruhe, Germany).

All oligonucleotides used in this study are listed in Supplementary materials (Tables A-E in S1 File) and were obtained from Eurofins-MWG-Operon (Ebersberg, Germany). All chemicals used were obtained from Sigma-Aldrich (Munich, Germany) unless otherwise stated.

### Identification of dimorphic switch deficient transformants

The transformants generated and wildtype reference strain IPO323 were spotted (1.5 μl; 1×10^8^ spores/ml) onto N-deprivation solid medium and water agar. Colonies were analysed stereomicroscopically using binocular Zeiss Stemi 2000-C (Carl Zeiss Microscopy GmbH) after 7, 14 and 21 days of incubation at 18°C to monitor the dynamics of dimorphic switch of strains generated. Morphology scoring was based on macroscopic colony appearance at 14 days, with consideration of appearance at 21 days in questionable cases. The mutants were considered as dimorphic switch deficient when their growth and switching inability was comparable with that of Δ*Zthog1* mutant strain as reference after 7 days post inoculation of N-deprivation and water agar medium.

### Nucleic acid manipulations

Unless otherwise specified, all DNA manipulations and molecular cloning procedures were carried out by standard protocols [27]. All restriction endonucleases and T4-DNA ligase were purchased from NEB. PCR amplification of fragments for cloning purposes was performed using Phusion High Fidelity Polymerase (New England Biolabs (NEB), Herts, UK). Diagnostic colony PCR was performed using Takara Sapphire Premix and all other PCR amplifications including those for genome walking were conducted using the Taq-Polymerase according to the manufacturer recommendations. Recombinant vectors were sequenced using the Sanger sequencing service at Eurofins Genomics (Ebersberg, Germany).

Plasmids were extracted from *E*. *coli* using the QIAprep spin mini-prep kit (Qiagen, Manchester, UK). Isolation of the genomic DNA from *Z*. *tritici* was routinely performed using Qiagen DNeasy Kit (Qiagen Sciences, Valencia, CA).

### Transformations using ATMT for the generation of random / targeted /complementation mutants

Transformation of conidia and selection of hygromycin-resistant transformants of *Z*. *tritici* were performed using *Agrobacterium tumefaciens*-mediated transformation (ATMT) [28,29]. *A*. *tumefaciens* strain AGL1, containing the constructed gene inactivation vectors based on pCAMB0380 (CAMBIA, Canberra, Australia), was cocultivated with conidia of *Z*. *tritici* for 48 hours at 28°C on AIM medium. Prior to this cocultivation the *Z*. *tritici* culture was grown for 3 days in YEG at 18°C and finally centrifuged for 10 min at 4000 rpm in order to harvest conidia and finally resuspend them to final concentration of 5×10^7^/ml. For random mutagenesis we used pCAMB-HPT(*Sal*I), a pCAMB0380-based vector, containing the hygromycin resistance gene *HPT* from pCB1003 [30] cloned as *Sal*I fragment into the *Sal*I site of the vector. The bacterial hygromycin B phosphotransferase (*HPT*) gene is expressed under the control of the strong constitutive *Aspergillus nidulans* trpC promoter, forming the *HPT*-cassette as selectable marker conferring the hygromycin resistance. For complementation of the mutant strains the vector pCAMB-BAR was used for ATMT. This vector is based on the plasmid pCAMB0380 and harbors a bialaphos resistance cassette cut with restriction enzyme *Sal*I from the plasmid pCB1635 and ligated into the plasmid pCAMB0380 restricted with *Sal*I. The detailed transformation procedure was previously described [31]. Selection of hygromycin resistant transformants containing the gene deletion constructs was performed using 200 mg/ml Hygromycin gold (Invivogen) on V8 agar, while selection of BASTA-resistant transformants produced within complementation of null mutants of *Z*. *tritici* was performed with 200 mg/l BASTA (glufosinate ammonium) on MM. Selection in both cases included 200 mg/l streptomycin and 350 mg/l cefotaxime.

### Pathogenicity assays

For pathogenicity tests, conidia were harvested from 4-day-old YEG submerged cultures and resuspended at 1×10^8^/ml conidia/ml in 0.2% gelatine. Ten-day old seedlings of the susceptible wheat line *cv*. *Riband* were used for plant infection assays by spray-inoculation. Inoculation, incubation and examination of intact plants were carried out as previously described [31]. The plants were examined 21 days post inoculation.

### Spore germination test

For the spore germination assay, conidia of *Z*. *tritici* strains were inoculated onto microscopic slides covered with a thin layer of water agar. For each strain 100 μl of spore suspension at final concentration of 10^6^/ml were spotted. The incubation was performed at 18°C in a moisture chamber with nearly full-saturated humidity for 72 h in the dark. The spores were directly microscopically analysed and the ratio of germination was calculated as the mean percentage of conidia germinated using three biological replicates for each strain.

### Spore lysis test

To investigate cell wall defects, mutant strains of *Z*. *tritici* and the wildtype reference IPO323 were compared under conditions which lead to lysis of the cell wall. The strains selected for examination were grown for 3 days at 18°C and 120 rpm in liquid medium YEG. The conidial cells were pelleted by centrifugation at 4,000 rpm and washed three times in an isotonic buffered solution (0.15 M Na_2_HPO_4_, 0.08 M citric acid, 1.4 M KCl; pH 5.8). Finally the spores were resuspended in the isotonic buffer containing 30 mg of lysing enzymes from *Trichoderma harzianum* (Sigma) by adjusting 10^7^ spores/ml for each strain investigated, followed by incubation for 3 h at 30°C with a slight stirring. The lysing rate was determined by enumerating the remaining intact yeast-like conidia (or filaments in case of *myco#5* mutant strain) using cell counting chamber (Neubauer improved, Carl Roth GmbH & Co.).

### Analysis and recovery of *HPT* integration sites within the random-mutant genome

The DNA from the site of the T-DNA insertion was recovered using a “step-down” PCR-based approach [26] by two nested rounds of amplification using the “gene-specific” primers gspBa, gspB, gspAa, gspA and “adapter-specific” primers PP1 and PP2 (Table B in S1 File). The amplicons obtained were finally cloned into pGEMTeasy or pJET vectors (Promega) and sequenced using universal primers (M13–20, T7 and M13 rev -49).

## Results

### Generation of a transformant library by ATMT and analysis of the mode of T-DNA integration into the genome of *Zymoseptoria tritici*

**Fig 1:**
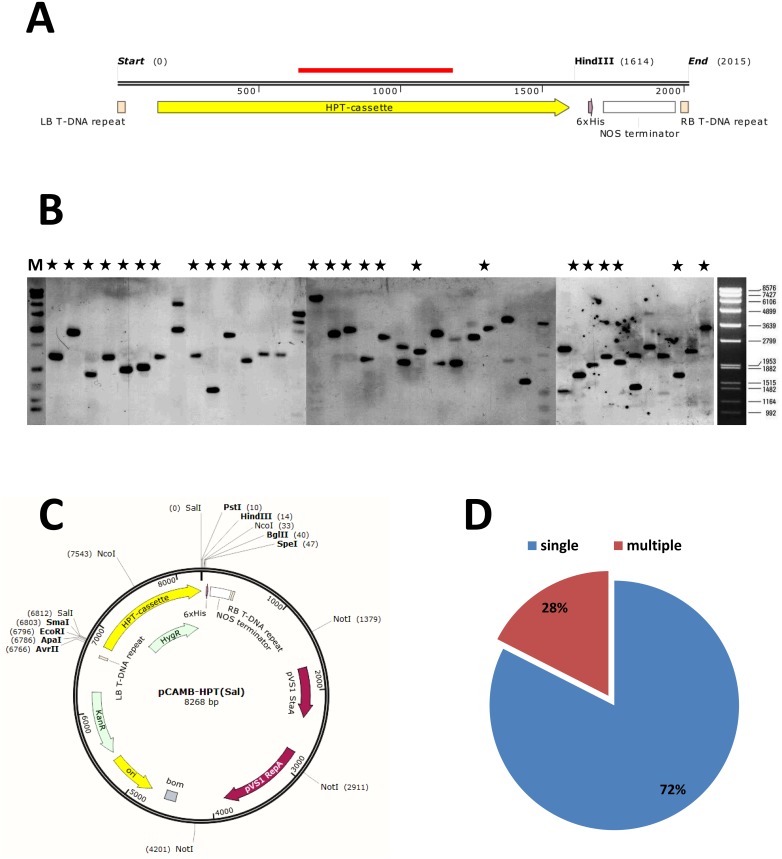
Analysis of *Z*. *tritici* random mutants generated by *Agrobacterium tumefaciens*-mediated transformation (ATMT). **(A)** Map of T-DNA construct used for genomic integration, containing *HPT* cassette (promoter+ORF+NOS terminator) flanked by left- and right-borders. *Hind*III restriction enzyme was used for the restriction of genomic DNA derived from the random mutants prior to the following Southern Blot analysis. The red bar indicates the location of probe used for hybridization. **(B)** Southern Blot analysis of 40 representative random transformants of *Z*. *tritici*. Genomic DNA of respective strains was restricted with *Hind*III. Black asterisks represent most likely a single T-DNA integration event; two or more hybridization signals indicate that most likely two or multiple T-DNA integrations in the recipient genome at different sites might have occurred. M: DNA Molecular Weight Marker VII, DIG-labeled (Roche Applied Biosciences). **(C)** Map of pCAMB-HPT (*Sal*I)-vector used for ATMT. **(D)** Pie chart showing the relative percentage of occurred T-DNA integration events in the generated random mutants (the estimated percentage refers to 100 randomly selected transformants).

Using an insertional random mutagenesis as a forward genetics approach, we generated a collection of 10,000 transformants of the wheat pathogen *Z*. *tritici*. The mutagenesis program was conducted by *Agrobacterium tumefaciens*-mediated transformation (ATMT), representing a stable and reliable transformation method, which was previously established and approved in different reverse genetics studies with *Z*. *tritici* [31]. pCAMB-HPT(*Sal*I) was used as a transformation vector (Fig 1C), containing hygromycin phosphotransferase gene (*HPT*) as a resistance marker derived from *Escherichia coli* under control of upstream *trpC* promoter from *Aspergillus nidulans* and downstream *NOS* terminator from *Agrobacterium tumefaciens* [31]. The suitability of random mutagenesis was proved by randomly selecting of 100 transformants and performing a Southern Blot analysis to verify the genomic integration of T-DNA and to assess the copy number of T-DNA. The isolated genomic DNA of the transformants which had been proved to be resistant to hygromycin B at 200 μg/ml, was fragmented with *HindIII* and probed with DIG-labelled *HPT*-fragment-probe (Fig 1A). This analysis revealed no bias towards multiple integrations of the T-DNA and confirmed a likely “randomized” distribution of integration sites, especially in view of the potential occurrence of so-called “hot spots” (which have been reported in the case of some fungi and plants). Overall, a nearly 72% frequency of single locus T—DNA integration had been observed with the site of insertion appearing to vary among transformants (Fig 1B and 1D).

### Screening and analysis of dimorphic switch deficient mutants

By cultivating the generated strains on nitrogen starvation medium, which represented the “dimorphic switch”-inducing medium by mimicking the natural condition encountered on the plant leaf, all the transformants were screened for reduced or complete inability to perform the yeast-to-hyphal transition. In a second round of screening the preselected transformants were assayed on water agar medium, also representing a strong switch-inducing condition in order to confirm the observed dimorphic switch deficiency of the generated transformants.

**Fig 2:**
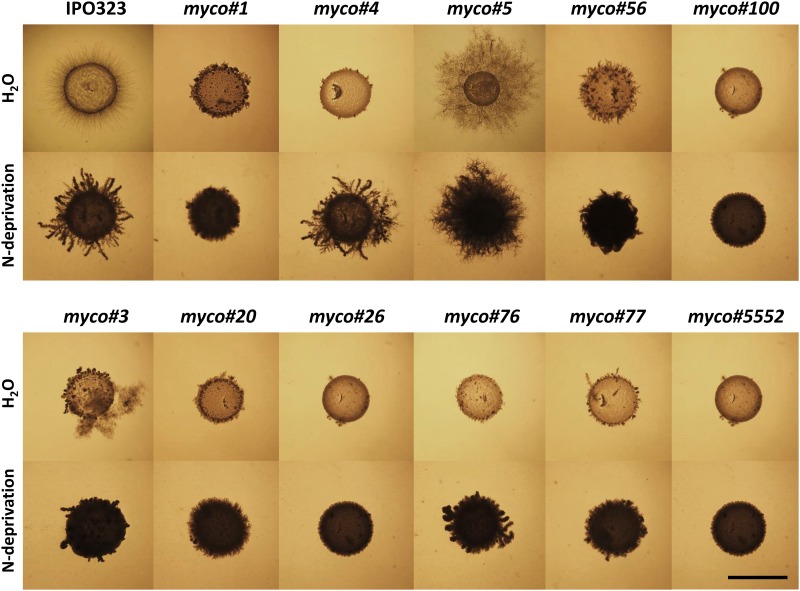
Analysis of *Zymoseptoria tritici* random mutant strains. The figure shows generated mutant strains (1.5 μl; 1×10^8^ spores/ml) grown on water agar medium and nitrogen starvation medium (N-deprivation) after 21 days of incubation at 18°C. N-deprivation medium was used as screening medium. Water agar (H_2_O), which provides no external nutrition source was used for second round of screening. The wildtype strain IPO323 grows filamentously and forms mycelium on N-deprivation medium and water agar medium. The strains *myco#1*, *myco#3*, *myco#20*, *myco#26*, *myco#56*, *myco#76*, *myco#77*, *myco#100* and *myco#5552* show an extreme reduction in mycelium formation on both screening media. The *myco#4* mutant grows poorly on H_2_O medium showing a drastically reduced mycelium formation, however being able to switch to filamentous growth on N-deprivation medium. In contrast, *myco#5* displays a hyperfilamentation phenotype under tested cultivation conditions, exhibiting a severely increased filamentous growth. Scale bar corresponds to 1 cm.

Thus, the outcome of the random mutagenesis approach resulted in 11 insertional mutants, exhibiting “dimorphic switch” deficiency, hence providing a recovery frequency of 0.11% when extrapolating from the total number of transformants screened. All of them remained mitotically stable, maintaining the hygromycin B resistance. Five successive passages of these transformants on YEG medium without hygromycin did not result in the loss of integrated T-DNA containing the *HPT* cassette. Thus, after five subcultures the transformants grew on V8 containing 200 μg/ml hygromycin B, suggesting that the *HPT* gene was stably integrated. The integration/insertion of *HPT* cassette was additionally confirmed in all mutants by colony-PCR (data not shown). Ten of the recovered mutants were growing predominantly yeast-like under “dimorphic switch”-inducing conditions (Fig 2).

Within the screening pipeline the dimorphic switch deficiency was defined as a qualitative measure rather than quantitative one, since across the experimental replicates for each of the mutant strains with reduced dimorphic transition ability were considerable fluctuations. Thus, none of the mutants (even the reference strain Δ*Zthog1*) completely failed to generate hyphae on inducing medium, indicating that dimorphic switch requires a complex and coordinated conditional expression of multiple genes or even gene networks. Although all of the mutants were drastically reduced in their ability of mycelium formation, some hyphal growth occurred occasionally, even in the Δ*Zthog1* used as reference strain. Thus, strains bearing a Δ*Zthog1*-like growth behaviour under switch-inducing condition after 7 days of incubation were considered as dimorphic switch deficient. Hence, nine random mutants, which were designated *myco#1*, *myco#3*, *myco#20*, *myco#26*, *myco#56*, *myco#76*, *myco#77*, *myco#100*, *myco#5552*, exhibited a yeast-like propagation under switch-inducing condition by incubating them on N-deprivation medium or water agar at 18°C (Fig 2). One mutant, designated *myco#5*, appeared to be locked in one growth stage producing a high amount of mycelium even on complete medium. Interestingly, *myco#3* appeared to produce slightly more mycelium after 14 days of incubation on water agar compared to other strains with a yeast-like growth behavior. In case of *myco#4*, one striking feature was apparent: within the first 7 days of cultivation on N-deprivation medium, the mutant strain grew exclusively yeast-like (data not shown). However, when following the prolonged incubation period of 21 days, the morphological conversion starting at 14 days of incubation from yeast-like towards filamentous growth was evident, indicating a delayed mode of transition compared to that of wildtype strain IPO323. Interestingly, when *myco#4* was grown on water agar, no visible formation of hyphae could be observed, even when the cultivation time was extended to 21 days. Furthermore, the growth was very sparse, resulting in a significantly reduced colony density compared to wildtype (Fig 2). For the random mutant *myco#5*, a predominantly filamentous growth was detected, regardless of the cultivation condition. Thus, incubating the mutant strain on the YEG standard medium at 18°C, which normally supports the yeast-like propagation of the wildtype strain, revealed in contrast to the wildtype a filamentous growth accompanied with the formation of aerial mycelium. Interestingly, a very similar growth behavior was noticed in YEG liquid medium, resulting in the enhanced mycelium formation, which was not observed in the wildtype strain IPO323 and other strains (data not shown).

Moreover, the results of the phenotypic screen with the selected mutant strains also agreed with pathogenicity assays on whole plants (susceptible cultivar Riband) as well as detached leaf assay. Generally, defects in dimorphic transition affected pathogenicity and resulted in very few necrotic flecks on the host plants. On the contrary typical disease symptoms (leaf necrosis and pycnidia formation) were observed on plants inoculated with conidial suspensions of the wildtype strain IPO323 (data not shown). A very intriguing phenotype was detected in the case of *myco#5*. Although this mutant predominantly grows filamentously, no typical symptoms of successful colonization of the plant developed.

### Molecular identification of T-DNA tagged gene loci

Genome walking analysis was carried out with all dimorphic switch deficient mutants in order to isolate DNA sequences flanking the T-DNA integration sites. Prior to genome walking analysis, the selected mutants were subjected to Southern Blot analysis to determine the T-DNA insertion copy number in their genomes. The isolated gDNA of each mutant strain obtained was restricted with endonucleases *Hind*III, *Psp*OMI and *Not*I (were all predicted to cut frequently in the genome sequence) and probed with PCR-amplified *HPT* cassette fragment. Hence, in case of *myco#1*, *myco#3*, *myco#4*, *myco#5*, *myco#26*, *myco#56*, *myco#76*, *myco#100* and *myco#5552* single hybridization signals were detected, indicating only single genomic sites of T-DNA insertion. For the mutant strains *myco#20* and *myco#77* several hybridization signals were observed, suggesting multiple genomic T-DNA insertion sites (data not shown).

**Fig 3:**
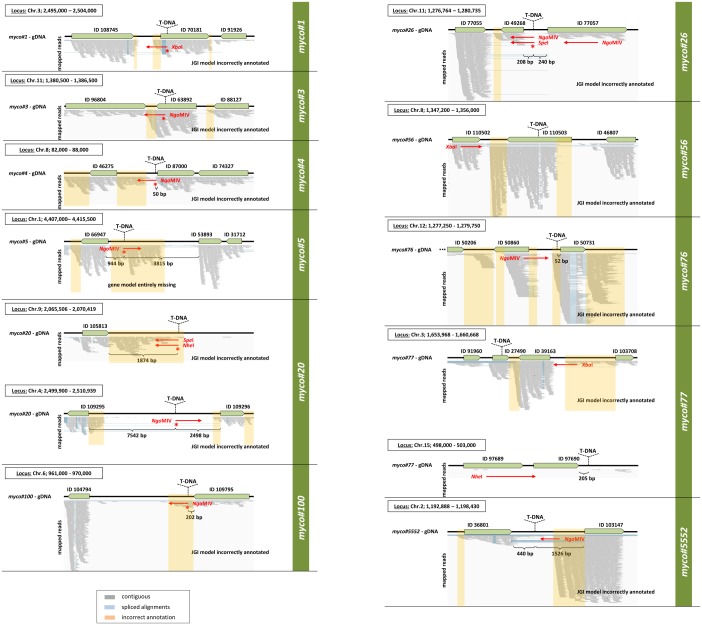
Results of genome walking analysis of recovered T-DNA disrupted gene loci. Predicted T-DNA insertion sites for each random mutant within putative genomic loci containing annotated genes are shown. DNA sequences obtained from genome walking PCR are illustrated as red lines aligned to a corresponding genomic locus; arrow heads determine the sequencing direction. Restriction enzymes used to obtain each of the DNA fragments prior to sequencing are indicated. Genes are named in concordance with JGI annotation by their Protein ID. The asterisks indicate the start position of the region matching the partial sequence of *HPT* cassette, thus pointing to the site of T-DNA integration for each mutant strain-specific locus. In case when the portion of *HPT* cassette was not identified, the putative genomic position of T-DNA inserted was estimated by fragment length obtained from restriction analysis and Southern Blot results. Predicted sites of T-DNA insertions are marked by vertical arrowheads. The “house-intern” RNA-Seq data of IPO323 were used to verify the current JGI models by mapping the aligned reads to the IPO323 genome sequence. The incorrectly annotated gene models obtained from JGI, as well as spliced junctions and contiguous sequences according to RNA-Seq data are indicated.

The T-DNA integration sites were recovered by “step-down”-PCR amplification of genomic DNA fragments acquired by endonuclease restrictions from two enzyme sets, followed by an adaptor ligation using a modification of the method previously described [26]. By interrogating the genome sequence of *Z*. *tritici* provided by JGI (Zymoseptoria Sequencing Project, Broad Institute of Harvard and MIT, http://www.broadinstitute.org/), these T-DNA flanking sequences allowed the localization of the T-DNA integration sites and identification of putative gene loci affected by these integration events (Fig 3).

Overall, among the T-DNA insertions sites detected, two (in case of *myco#3* and *myco#56*) were located inside predicted open reading frames (ORFs) of annotated genes, while the remaining nine genes (*myco#1*, *myco#4*, *myco#5*, *myco#20*, *myco#26*, *myco#76*, *myco#77*, *myco#100* and *myco#5552*) were positioned in the intergenic regions, meaning upstream or downstream of predicted ORFs. In addition, we used the “house-intern” (unpublished) RNA-Seq data derived from IPO323 cultures grown for 7 days under dimorphic switch inducing condition (N-deprivation medium), as well as publically available RNA-Seq data (obtained from Sequence Read Archive (SRA) at NCBI), to evaluate the existing gene models obtained from JGI. For most of the genes the predicted models were found to be incomplete (Fig 3). In case of *MYCO5*-locus, one gene model appears to be entirely missing, however there was a clear evidence of transcription based on RNA-Seq read alignments. For the remaining gene loci obtained, the boundaries of the JGI gene models were found to exclude the full extent of the transcribed regions. Hence, for e.g. *MYCO76* and *MYCO100* the entire exons of the corresponding genes are missing (Fig 3). The JGI database IDs for the predicted genes derived from genome walking analysis as well as further information concerning homology analysis and genomic coordinates of T-DNA insertions sites are shown in Table 1. The following section describes the random mutants for which T-DNA insertion sites could be identified:

**Table 1:** Summary of *Z*. *tritici* genes identified from T-DNA flanking sequences.

Mutant strain	T-DNA location / Protein ID (JGI)	Size nt (aa)	BLASTp match with functional annotation	Organism	BLASTp E-value (identity %)
***myco#1***	**Chr.3 at position 2.499.750 / ID: 70181**	**2277 (589)**	two-component response regulator SSK1p	[*Blumeria graminis* f. sp. hordei DH14]	**1e-156 (52%)**
***myco#3***	**Chr.11 at position 1.383.695 / ID: 63892**	**1125 (373)**	Hypothetical protein (letm1-like protein)	[*Marssonina brunnea* f. sp. 'multigermtubi' MB_m1]	**1e-37 (37%)**
***myco#4***	**Chr.8 at position 84.634 / ID: 87000**	**1343 (428)**	phosphoribosylamine-glycine ligase	[*Sphaerulina musiva* SO2202]	**0.0 (72%)**
***myco#5***	**Chr.1 at position 4.409.323 / ID: 66947**	**1441 (247)**	Hypothetical protein (Modifier of rudimentary)	[*Macrophomina phaseolina* MS6]	**2e-55 (53%)**
	**Chr.1 at position 4.409.323 / no ID**	–	Hypothetical protein (TATA element modulatory factor 1, TATA binding protein)	[*Macrophomina phaseolina* MS6]	**1e-124 (40%)**
***myco#20***	**Chr.4 at position 2.507.660 / ID: 109296**	**1790 (551)**	amino acid permease	[*Trichophyton rubrum*]	**1e-154 (54%)**
	**Chr.9 at position 2.068.569 / ID: 105813**	**671 (120)**	Hypothetical protein (*Z*. *tritici* unique)	–	–
***myco#26***	**Chr.11 at position 1.278.735 / ID: 49268**	**498 (165)**	Hypothetical protein (DUF1768-domain containing protein)	[*Alternaria alternata*]	**2e-39 (44%)**
	**Chr.11 at position 1.278.735 / ID: 77057**	**1730 (480)**	Zinc finger RING protein	[*Macrophomina phaseolina* MS6]	**8e-87 (36%)**
***myco#56***	**Chr.8 at position 1.351.161 / ID: 110503**	**2931 (743)**	RNA-binding post-transcriptional regulator cip2	[*Aspergillus lentulus*]	**4e-166 (54%)**
***myco#76***	**Chr.12 at position 1.278.989 / ID: 50731**	**310 (76)**	DUF1242-domain-containing protein	[*Sphaerulina musiva* SO2202]	**1e-39 (88%)**
***myco#77***	**Chr.3 at position 1.655.698 / ID: 27490**	**600 (200)**	copper homeostasis protein CutC	[*Flavobacterium cauense*]	**6e-48 (53%)**
	**Chr.15 at position 502.425 / ID: 97690**	**1524 (434)**	Hypothetical protein (*Z*. *tritici* unique)	–	–
***myco#100***	**Chr.6 at position 967.563 / ID: 109795**	**2133 (582)**	Patatin/Phospholipase A2-related protein	[*Macrophomina phaseolina* MS6]	**2e-139 (51%)**
***myco#5552***	**Chr.2 at position 1.195.392 / ID: 36801**	**1258 (396)**	Methionine synthase, vitamin-B12 independent	[*Purpureocillium lilacinum*]	**0.0 (75%)**
	**Chr.2 at position 1.195.392 / ID: 103147**	**1054 (289)**	Delta(12) fatty acid desaturase	[*Pyrenophora tritici-repentis* Pt-1C-BFP]	**3e-134 (70%)**

The gene and protein sequences were retrieved from the *Z*. *tritici* completed genome v.2 database at http://genome.jgi-psf.org/Mycgr3/Mycgr3.home.html. For each random mutant the T-DNA insertion site identified by genome walking approach as well as the best BLASTp match with provided E-value and corresponding organism are shown.

#### myco#3

In the random mutant *myco#3*, the T-DNA integration occurred in ORF of the gene predicted to encode protein of LETM1 family. LETM1 domain (leucine–zipper–EF hand-containing transmembrane region) containing proteins represent mitochondrial proteins conserved in all lower eukaryotes, animals, and plants [32].

#### myco#5

In case of *myco#5*, sequence analysis of the *HPT*-insertion region using BLASTn against the genome sequence of *Z*. *tritici* (provided by JGI server) revealed that T-DNA was integrated into the promoter region of the annotated gene with JGI Protein ID 66947 (MYCGR3_66947). This gene is predicted to encode a hypothetical protein comprising a *mod_r* domain (PF07200). The presence of *mod_r* domain suggests an involvement of this protein in endosomal trafficking. At the same time, the presence of the aligned reads in this region indicates however the existence of one further gene being missing in the current JGI genome annotation (Fig 3). BLASTn interrogation of the corresponding ORF sequence of the missing gene against the non-redundant protein sequence (nr) NCBI database yielded a hypothetical TATA binding protein encoding gene as the best search hit. The location of the T-DNA integration would suggest an interruption of the ORF, leading to the conclusion that either the newly identified gene or MYCGR3_66947, or both of them in additive manner might be causative for the mutant’s phenotype observed.

#### myco#20

For *myco#20* the genomic T-DNA location was found on two different chromosomes, thus indicating a multiple mode of integration. However, in both cases a relatively large distance to the nearest annotated neighbor genes encoding hypothetical proteins was apparent. Interestingly, when considering the RNA-Seq data, the T-DNA integration predicted on chromosome 9 was found in the nearest proximity to the gene with Protein ID 105813 (Fig 3). Since the RNA-Seq reads aligned were found in the downstream region of the gene, the gene model proposed by JGI appears to be inaccurately annotated.

#### myco#26

Identification of T-DNA insertion in *myco#26* mutant revealed two potential genes affected, both of which are suggested to be responsible for the observed phenotype of the mutant, since the integration site is located within promoter regions of both genes. For the first gene (Protein ID 49268), no functionally characterized homologs from other organisms were found when performing the BLASTp analysis. However, the second one (Protein ID 77057) was found to encode a protein belonging to RING zinc finger protein family, indicating its role as a potential transcription factor.

#### myco#56

In the *myco#56* random mutant, T-DNA integration occurred within the ORF of the gene with JGI Protein ID 110503 (MYCGR3_110503). The most similar previously characterized protein found in the NCBI “nr”-database was the *Schizosaccharomyces pombe* protein Cip2p (Acc. No. NP_592895.1; E-value: 3e-41), which shares 32% of its amino acids with Myco56p. This *S*. *pombe* protein homolog was previously functionally characterized and shown to interact with Csx1p, which in turn controls global gene expression during oxidative stress in *S*. *pombe* by regulating the turnover rate of *ATF1* transcripts [33,34].

#### myco#76

Meanwhile, in random mutant *myco#76*, the T-DNA insertion was identified in the promoter region of the gene encoding a hypothetical protein harboring *DUF* domain. The RNA-Seq data revealed, however, reads mapped in the upstream region, indicating that T-DNA insertion could affect the gene expression by interrupting the exon or interfering with 5’-UTR region missed in the current JGI annotation (Fig 3).

#### myco#77

One further dimorphic switch deficient mutant assigned *myco#77* was found to be disrupted either in gene with no functional annotation located on chromosome 3 or gene on dispensable chromosome 15 predicted to encode copper (Cu) homeostasis protein CutC. The corresponding T-DNA insertion sites have been detected in ORF of the gene MYCGR3_27490 and terminator region of the annotated gene MYCGR3_97690, respectively.

#### myco#100

In case of *myco#100*, a Patatin/Phospholipase A2-related protein encoding gene was affected by T-DNA disruption. The T-DNA insertion was found to be located in the terminator region (appr. 200 bp downstream of the ORF annotated). However, when considering the RNA-Seq data for annotation evaluation, the T-DNA insertion occurred in the postulated exon region within the ORF of the gene being yet not correctly annotated (Fig 3).

#### myco#5552

In the random mutant *myco#5552* the integration site of T-DNA occurred in chromosome 2 between the annotated genes potentially encoding Δ12-fatty acid desaturase (FAD) and methionine synthase, respectively. Also in this case we could state the incorrectly annotated gene with ProteinID 103147, harbouring the transcribed region upstream of the annotated ORF (Fig 3).

The remaining two mutant strains designated ***myco#1*** and ***myco#4*** were selected for further detailed investigation within this study. Similarly to the other genes mentioned above, the corresponding gene models predicted in both genomic loci were evaluated by employing the gene fitting comparison with the RNA-Seq data. For the *MYCO1* gene locus, the T-DNA insertion was retrieved in the exon region of the gene with Protein ID 70181, in contrast to the initial assumption of T-DNA inserted in the promoter region according to JGI annotation. In case of *MYCO4* locus the T-DNA insertion site was assigned to a transcribed upstream region of the inaccurately annotated gene with Protein ID 87000 (Fig 3). Both predicted genes (*MYCO1* and *MYCO4*) for which the mutants were suggested to be disrupted were functionally characterized exploiting reverse genetics approach by targeted gene inactivation.

### Verification of random mutants by targeted gene inactivation and initial phenotypic characterization

Thus, to deliver a proof of concept and to validate the genome walking results, we selected two genes for inactivation, designated *MYCO1* and *MYCO4*, the orthologs of which were previously characterized in other fungal organisms.

**Fig 4:**
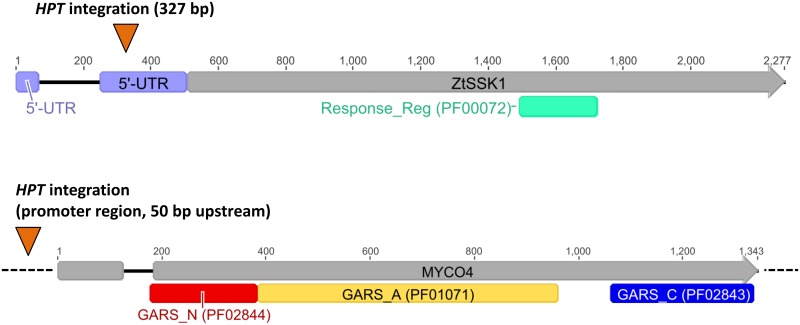
Structural analysis of target genes. The figure illustrates the structure of the predicted target genes *ZtSSK1* (A) and *MYCO4* (B) affected by random mutagenesis. T-DNA location sites (*HPT*-integration) assigned in the corresponding gene loci are indicated. The numbers over the bars indicate the length of the annotated genes according to JGI gene models. The highlighted bars represent the functional domains of each gene, determined by Pfam database interrogation using InterProScan and mapped to the corresponding gene model. Introns are indicated as black lines joining gene exons, the latter represented as gray bars. Dash lines depict flanking regions of the genes. Orange triangles mark identified position of T-DNA insertion for each target gene. UTR: untranslated region.

Hence, a detailed sequence analysis of the amplified flanks in case of mutant *myco#1* revealed that the T-DNA integration occurred in the 5’-UTR region (at 327 bp according to JGI gene model) or in the predicted exon region (when considering the RNA-Seq data) of the annotated gene with the Protein ID 70181 according to JGI Institute database or MYCGR3_70181 according to Ensemble (Fig 4). The precise location of T-DNA insertion was determined on chromosome 3 at genomic position 2.499.750 (Table 1, Fig 3). BLASTp analysis of the deduced protein sequence indicated that this gene encodes a homolog of Ssk1p, a known regulator within the *HOG1*-pathway in fungi [35–37], based on similarity to the *Magnaporthe oryzae* homologous protein with significant E-value of 1.24e-153 and 99% identity. Myco1p is a predicted 589 amino acid protein harboring a *Response_Reg* domain (Pfam ID: PF00072) located between amino acids 329–406. Based on these finding we designated the gene *ZtSSK1*.

For the *myco#4* mutant, the genomic T-DNA insertion location was identified on chromosome 8 at genomic position 84.634 (Figs 3 and 4), about 50 bp upstream of a predicted gene (JGI Protein ID: 87000; Ensembl: MYCGR3_87000) with high similarity to the bifunctional *Saccharomyces cerevisiae* ADE5,7 (46% amino acid identity) required for the second and fifth step of *de novo* purine nucleotide biosynthesis (Table 1). When considering the information obtained from RNA-Seq data, the T-DNA insertion is suggested to interrupt the hypothetical 5’-UTR region of the gene. This gene was previously functionally characterized in the yeast *S*. *cerevisiae* and *C*. *albicans* encoding a bifunctional enzyme possessing aminoimidazole ribotide synthase and glycinamide ribotide synthase activities [38,39]. The similarity is largely confined to the region where the predicted functional domains are located. These domains include *PRibGlycinamide_synth_N* (PF02844), *PRibGlycinamid_synth_ATP-grasp* (PF01071) and *PRibGlycinamide_synth_C-dom* (PF02843). However, using alignment analysis, we noticed an absence of functional domains *AIRS* (PF00586) and *AIRS_C* (PF02769) typically present in other fungi including the yeast homolog, indicating that the *Z*. *tritici* counterpart is monofunctional (Fig B in S1 File). In the pre-screening the random mutant lacking the functional gene exhibited a poor vegetative colony growth on poor-nutrient media like N-deprivation and water agar, providing a nutrient-less medium. When grown on YEG standard complex medium a very similar phenotype to wildtype was observed. Also, no defects in spore morphology were visible when comparing to the wildtype strain IPO323. However when grown on MM or N-deprivation medium the colonies of *myco#4* mutant exhibited a sparse growth compared to wildtype, prohibiting a considerable biomass increase, thus indicated auxotrophic properties of the mutant.

The two genes selected were successfully inactivated through replacement of their ORF-portion of with *HPT* cassette conferring hygromycin resistance (Figs C and D in S1 File). The physiological properties of generated targeted mutants (in the following indicated as Δ*Ztssk1* (= Δ*myco1*) and Δ*myco4*) were confirmed exhibited the same phenotype as the corresponding random transformants. Genetic complementation of the targeted inactivation mutants with the corresponding native gene loci also demonstrated the responsibility of the respective genes for the observed phenotype, as far as the wildtype phenotype could be recovered. Therefore, the mutation resulting in the observed phenotypes corresponded to the loci for which the sequence was obtained as the T-DNA flank. As expected, on the switch-inducing medium (N-deprivation medium), which was used for the screening of switch-deficient transformants, Δ*Ztssk1* and Δ*myco4* showed an impaired ability for filamentous growth. This observation was also supported by results obtained from germination assays (Fig A(B) in S1 File). In the case of Δ*myco4* the yeast-to-hyphal transition was observed, but it appeared in a delayed manner after two weeks of incubation under defined conditions in comparison to the wildtype-strain where transition was visible after one week of incubation. Furthermore growth of Δ*myco4* under nutrient deprivation provided by N-deprivation and H_2_O agar media was found to be reduced and resulted in a poor colony development associated with a reduced cell density. However incubation of the mutant on YEG restored these growth defects and a very similar phenotype to that of wildtype was apparent. On the water agar both mutants were found to grow yeast-like, even if the incubation period was prolonged up to 21 days.

### Dimorphic switch as determinant factor for full pathogenicity

**Fig 5:**
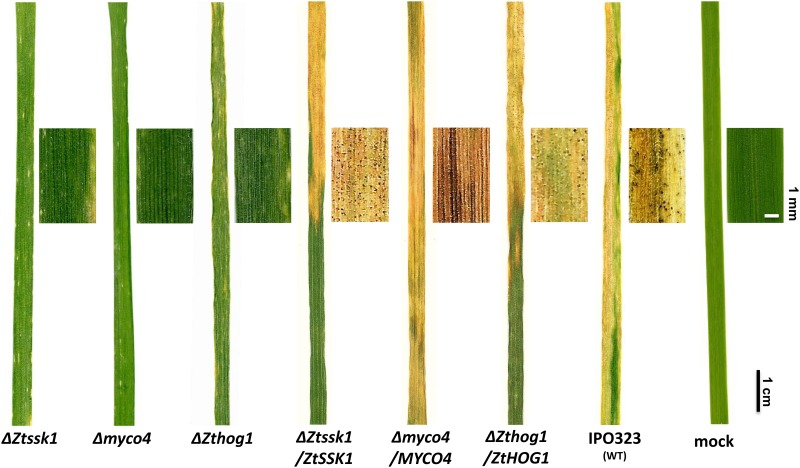
Plant infection assay with selected mutant strains. Effects of the gene inactivation on disease development in the susceptible wheat cv. Riband after 21 days after inoculation (21 dpi) are shown. WT IPO323 and Δ*Zthog1* are regarded as references and mock as negative control, meaning plant leaves were sprayed with water only. The incubation of infected plants was performed with the following parameters: temperature: 22°C, humidity: 80%, Lightcycle: 16 h / 8 h. Both Δ*Ztssk1* and Δ*myco4* along with Δ*Zthog1* are drastically reduced in virulence. Meanwhile, typical symptoms of successful infection were observed for WT IPO323, forming mature pycnidia 21 dpi. Full virulence to the loss-of-function mutants was completely restored by retransformation of the strains with respective functional full-length gene copies.

In order to verify whether dimorphic switch deficiency impacts the pathogenicity of the mutants, we inoculated the susceptible wheat cultivar Riband with the targeted mutant strains and IPO323 along with Δ*Zthog1* as the reference strain. Expectedly, we didn’t observe any symptoms of typical STB disease on plants inoculated with Δ*Ztssk1* and Δ*myco4*. Both developed only small chlorotic lesions, which were also partly noticed for mock control, indicating the senescence and aging of the host plants (Fig 5). In constrast, the wildtype developed at first chlorotic and later necrotic lesions in course of disease progression and formed pycnidia after 21 days post inoculation. Similar results were obtained for complemented strains Δ*Ztssk1/ZtSSK1* and Δ*myco4/MYCO4* generated by reintegration of intact gene copy for which the targeted mutants were disrupted. For both complemented strains the wildtype-like virulence was recovered and after latent phase of infection they finalized their infection cycle by formation of pycnidia bearing asexual spores (Fig 5).

### Δ*Ztssk1* is sensitive to osmotic and oxidative stress

**Fig 6:**
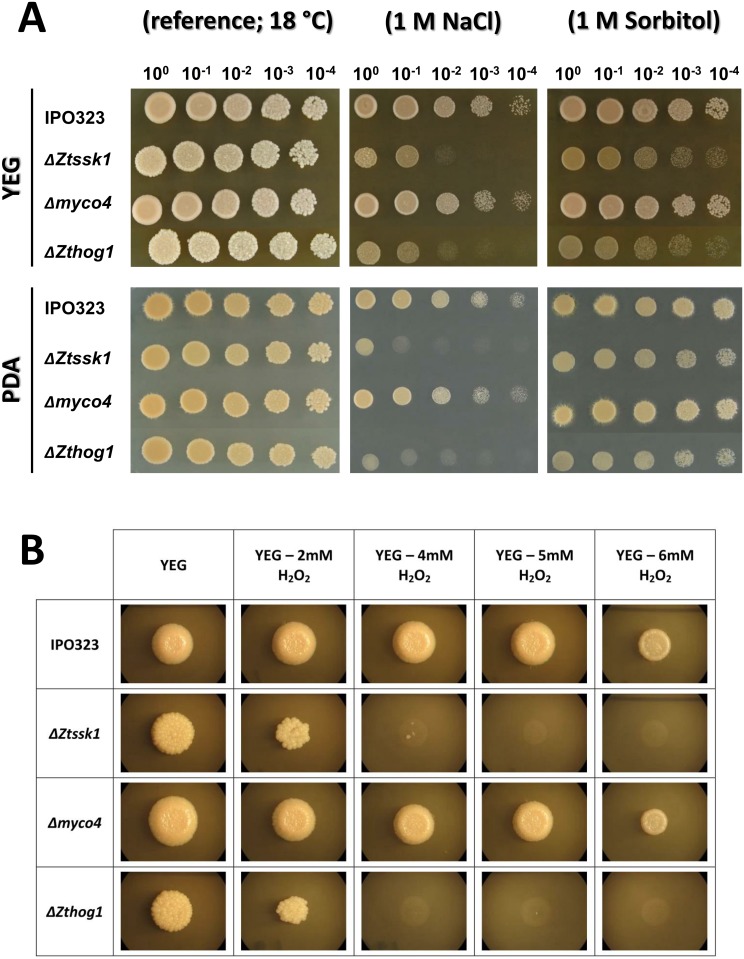
(**A)** Influence of inactivation of target genes on growth under osmotic stress. Spores of the corresponding mutant strains shown were spotted on the indicated media in serial dilutions (1.5 μl; 10^8^ spores/ml). The plates were incubated for 7 days at 18°C. The phenotypes observed were verified by three independent experiments. (**B)** Examination of susceptibility of generated mutant strains to oxidative stress. Colony morphology of *Zymoseptoria tritici* IPO323 and generated mutants under oxidative stress growth conditions. The wildtype (WT) strain and indicated mutant strains of YEG cultures were spotted as dilution series (1.5 μl; 5×10^7^ spores/ml) and incubated on YEG plates containing various concentrations of H_2_O_2_. Plates were imaged after 7 days of incubation at 18°C. Strains lacking *ZtSSK1* or *ZtHOG1* are more susceptible to oxidative stress, compared to the rest.

To assess the implication of ZtSsk1p in canonical osmoregulation consistent with its role as a HOG-pathway constituent, the Δ*Ztssk1* mutants as well as wildtype and Δ*myco4* as controls were incubated on PDA and YEG media supplemented with NaCl and sorbitol at different concentrations. Both, Δ*Ztssk1* and Δ*Zthog1* were drastically reduced in growth under all osmotic stress conditions tested compared to control strains, however less pronounced when incubating upon sorbitol stress (Fig 6A). As expected, the growth of Δ*Zthog1* was impaired compared to wildtype after 7-day incubation on YEG or PDA supplemented with 1 M sorbitol/NaCl, resulting in reduced conidial density. A very similar phenotype to that of Δ*Zthog1* was observed in the case of Δ*Ztssk1*, which was accompanied by a drastic growth reduction on the agar media mentioned above. Moreover, both were found to lack the ability to yeast-to-hyphal transition when incubating on PDA amended with osmotic stressors. This was in contrast to wildtype, Δ*myco4* and complementation strains exhibiting normal growth and dimorphic switch ability (Fig 6A).

Furthermore, we tested the sensitivity to oxidative stress consistent with evidenced implication of HOG-pathway in oxidative stress response provided by previous studies with other fungal organisms. Hence, incubating both Δ*Zthog1* and Δ*Ztssk1* on YEG supplemented with hydrogen peroxide even at low concentration (4 mM) resulted in the anticipated sensitivity consonant with results previously described [21]. Both exhibited a considerably reduced growth in contrast to Δ*myco4* and wildtype strain IPO323 as a control, displaying only a slightly reduced growth (Fig 6B).

### Disruption of *ZtSSK1* results in fungicide resistance

**Fig 7:**
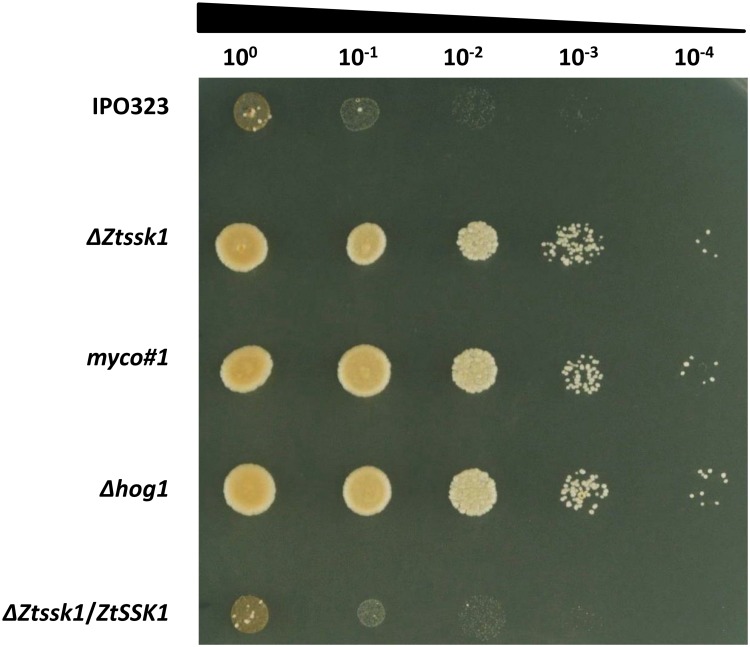
*In vitro* fludioxonil fungicide sensitivity assay. Prior to photographs the strains were incubated on PDA amended with fludioxonil (30 μg/ml, dissolved in EtOH) for 7 days at 18°C. Four times serial dilutions of the YEG-grown culture (1×10^8^ spores/ml) were dropped on the plate. The Δ*Ztssk1*, Δ*Zthog1* as well as random *myco#1* mutants display resistance to the phenylpyrrole fungicide fludioxonil. Meanwhile, the wildtype strain IPO323 and the ectopic transformant exhibit abolished growth upon fludioxonil treatment.

To investigate the role of the response regulator ZtSsk1p in fungicide sensitivity, *in vitro* growth assays were performed. The outcome of the test revealed that *ZtSSK1* disrupted mutants along with Δ*Zthog1* strains maintained an increased resistance to phenylpyrrole fungicide fludioxonil, both exhibiting similar phenotypes (Fig 7). This observation coincides with previous reports by Mehrabi *et al*., showing resistance of *ZtHOG1* deficient mutant strains against phenylpyrrole fungicides, fludioxonil and fenpiclonil, as well as dicarboximide fungicide iprodione [21]. Contrarily, incubation of IPO323 reference strain and complementation strain Δ*Ztssk1*/*ZtSSK1* on PDA supplemented with 30 μg/ml fludioxonil resulted in a strongly pronounced sensitivity against fludioxonil. This observation also coincides with previously reported results with *Neurospora crassa*, *Colletotrichum lagenarium* and *Alternaria alternata*, indicating that ZtSsk1p acts upstream of ZtHog1p and both contribute to fungicide sensitivity [40–42].

### *ZtSSK1* and *ZtHOG1* are required for proper morphology and cell wall biogenesis

Furthermore, Δ*Ztssk1* mutants were found to be affected in their morphology by forming partly ovoid/swollen conidia (Fig A(A) in S1 File). Conidia prepared from Δ*Ztssk1* mutants germinated at rates and magnitudes much slower than those of wildtype when assayed on microscopic slides covered with water agar. More than 80% of wildtype conidia germinated while less than 12% of conidia prepared from Δ*Ztssk1* mutants were able to form germ tubes (Fig A(B) in S1 File). Moreover, the length of germ tubes produced from mutant conidia was significantly shorter than those of wildtype. Conidia collected from wildtype were found to predominantly form multiple germ tubes. In contrast, the majority of Δ*Ztssk1* mutant conidia produced single germ tubes, indicating that mutation of *ZtSSK1* affected the pattern of conidial germination (data not shown).

**Fig 8:**
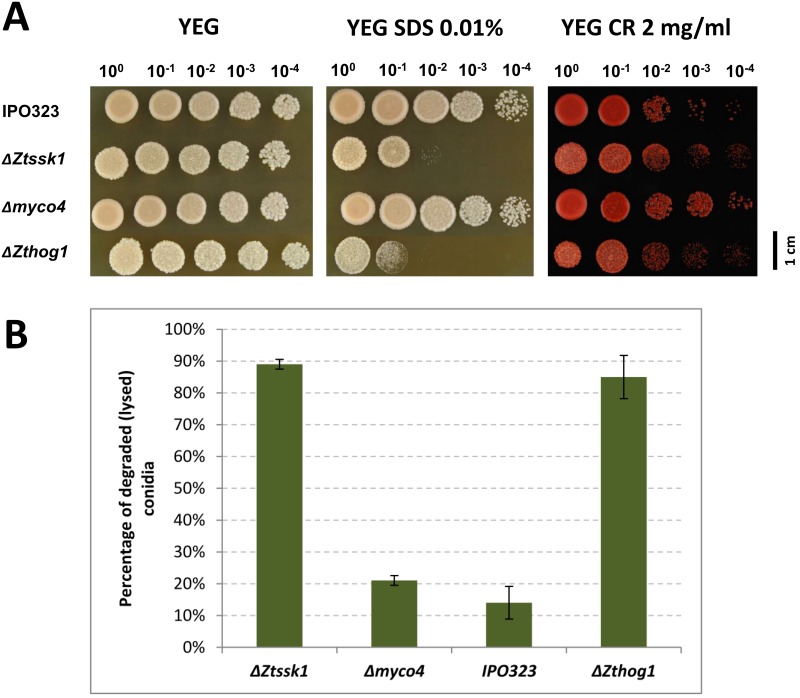
Involvement of the target genes *ZtSSK1* and *MYCO4* in maintaining the cell wall integrity. **(A)** Sensitivity of mutant strains to cell wall perturbing agents. The reference and mutant strains were grown on YEG medium, supplemented with 0.01% SDS or 2 mg/ml Congo Red (CR), for 7 days at 18°C. Four times serial dilutions of the grown culture (1×10^8^ spores/ml) were dropped on the plate. (**B)** Lysis test to examine protoplast release of the wildtype IPO323 and generated mutant strains. Percentage of degraded conidia due to protoplast production of corresponding strains is shown. The mutant strains Δ*Ztssk1* and Δ*Zthog1* display an enhanced sensitivity towards treatment with cell wall degrading enzymes from *Trichoderma harzianum* compared to wildtype. Data presented are the mean (including standard deviations) of results from the 3 independent experiments.

Furthermore, to investigate whether the mutants exhibit cell wall defects, the wildtype and Δ*Ztssk1* were plated on media containing cell wall / membrane perturbing agents Congo red and sodium dodecyl sulfate (SDS). Congo red interferes with cell wall by binding β (1–3) glucan, whereas SDS is known to disrupt the plasma membrane. Compared to the wildtype, the Δ*Ztssk1* strains showed increased sensitivity to SDS, indicating that *ZtSSK1* is involved in plasma membrane biogenesis, whereas the sensitivity to Congo red was unaffected (Fig 8A). Moreover, compared to wildtype the strains Δ***Zt****ssk1* and Δ***Zt****hog1* were found to be frequently lysed when treated with CWDEs (Fig 8B). Neither Δ*Ztssk1* nor Δ*Zthog1* treated with CWDEs was able to generate viable protoplasts, forming numerous broken cell fragments (data not shown) indicative of cell autolysis.

### Mutant lacking *MYCO4* gene exhibits adenine auxotrophy

**Fig 9:**
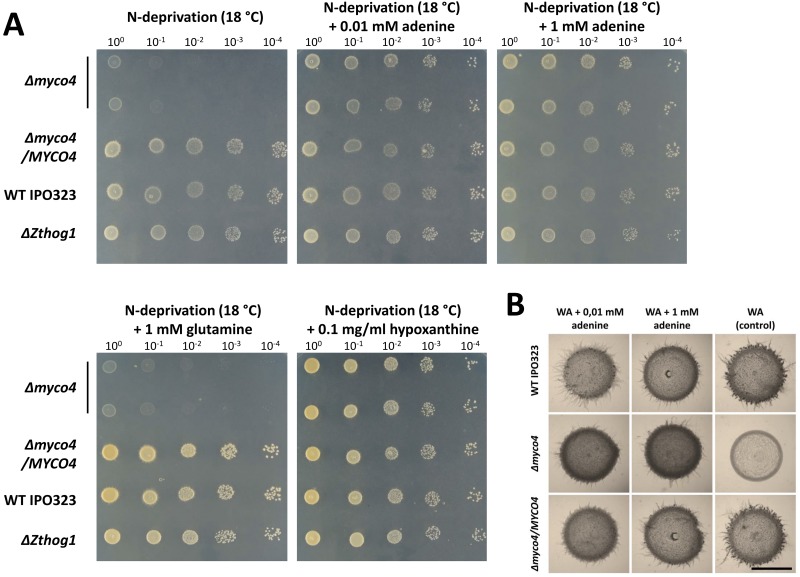
Growth of the Δ*myco4* mutant under purine auxotrophic conditions. **(A)** Inactivation of *MYCO4* gene results in the auxotrophic, purine requiring mutant. *MYCO4* deficient mutant is unable to grow on media, lacking the external source of adenine or hypoxanthine. Complementation of Δ*myco4* mutant with the full-length copy of *MYCO4* gene including its native promoter and terminator region or incubation of Δ*myco4* in the presence of 0.01/0.1 mM adenine or 0.1 mg/ml hypoxanthine restored the wildtype growth. The examination of colony growth and morphology was performed after 7 days incubation on respective media at 18°C. The isolated spores were spotted as dilution series (1.5 μl; 10^8^ spores/ml). **(B)** Yeast-to-hyphal transition restored for Δ*myco4* when incubating on water agar (WA) supplemented with 0.01 mM or 1 mM adenine for 7 days at 18°C.

Proceeding from the previous BLASTp analysis with the *MYCO4* gene revealed its involvement in *de novo* purine biosynthesis, insofar as significant functional orthologs previously characterized in other fungi were identified. Consistent with the putative role for *MYCO4* gene in the *de novo* purine biosynthesis, the incubation of *MYCO4* defective mutant on N-deprivation medium without adenine resulted in a drastically impaired growth (Fig 9A). As expected, Δ*myco4* also failed to grow on N-deprivation solid medium supplemented with 1 mM glutamine (Fig 9A), as well as glycine and aspartate (data not shown), which are normally required as precursors for *de novo* synthesis of purine nucleotides. Supplementation of N-deprivation medium with both, either adenine or hypoxanthine at 1 mM, restored the wildtype-like phenotype of the mutant completely, suggesting that the purine salvage pathway is operational in the mutant strain. The growth was also restored and was comparable to that of the wildtype strain when the native copy of the *MYCO4* gene was reintroduced in the loss-of-function mutant, indicating that the growth defect of Δ*myco4* was due to the lack of functional *MYCO4* allele. Interestingly, using lower concentrations of adenine added to N-deprivation medium the growth defect of Δ*myco4* mutant could be rescued and the colony development was similar to that observed for the wildtype strain IPO323. Furthermore, there was no difference in growth when Δ*myco4* was incubated on N-starvation medium supplemented with higher concentration of adenine or hypoxanthine, suggesting a sufficient uptake of external purines from the medium. The same phenotype was observed by cultivating the Δ*myco4* mutant strain on N-starvation medium supplemented with reduced amount of hypoxanthine (Fig 9A). Furthermore, as previously mentioned, the yeast-to-hyphal transition of Δ*myco4* occurred after a lag period of approximately 14 days on N-deprivation medium and was completely abolished on water agar. With addition of external adenine, even at low concentration, the wildtype-like “dimorphic switch” ability of the mutant strain was restored (Fig 9B). Consequently, these observations indicate that *MYCO4* is involved in *de novo* purine biosynthesis in *Z*. *tritici*, since the loss-of-function allele causes auxotrophic requirement for adenine.

## Discussion

To employ novel fungicides-based control strategies in order to combat STB, there is a clear need to understand the fine tuning infection mechanisms implemented by *Z*. *tritici* to colonize the host plant. Elucidation of molecular key determinants governing the pathogenic development of the fungus is crucial for effective biological control. Dimorphic switch represents one of the key features of *Zymoseptoria tritici*. Generally, dimorphism represents an important therapeutic target of dimorphic fungi, considering not only phytopathogenic representatives but also human systemic fungi, enabling invasion/penetration of the host organism [43–45]. Ability to promote the dimorphic transition is an essential prerequisite for pathogenesis. However, the molecular mechanisms governing this important critical process are not well understood and require further in depth analysis. In this study we aimed to identify and characterize the function of new dimorphism-related factors from *Z*. *tritici* regulating dimorphic transition.

To this end, *Agrobacterium*-mediated insertional mutagenesis was used to generate a collection of approximately 10,000 random mutants to identify novel genes required for dimorphic transition. Generally, insertional mutagenesis screen represents an effective forward genetics approach allowing discovering of novel genes in an unbiased manner. This stays in contrast to reverse genetics approach, which is based on the prior knowledge of the gene to investigate. In this view, the random mutagenesis aims to achieve an efficient random disruption of the genome and subsequent retrieval of tagged genes from the mutant of interest [46]. ATMT has been exploited extensively in random mutagenesis experiments to identify genes involved in the pathogenic interactions of fungi with plants, insects, mammals and other fungi [47–49]. In our experiment, nearly 70% of the transformants had single-site T-DNA integrations, an observation similar to that observed in previously reported experiments with *Magnaporthe oryzae* [50] and *Colletotrichum graminicola* [51]. A systematic analysis of T-DNA insertion pattern in 100 independent transformants revealed that the distribution of T-DNA inserts occurred likely randomly, precluding the biased integration in so-called “hot spot” regions previously reported for some fungi and plants. Since dimorphic switch represents an essential feature of the fungus to invade the host plant, we screened the generated mutants for switch deficiency. With this approach we isolated 11 random mutant strains, lacking the ability to undergo dimorphic transition under switch-inducing condition. Using genome walking the T-DNA disrupted gene loci were identified. Relying on RNA-Seq data, most of the gene models in the gene loci of interest appeared to be incorrect, indicating that gene annotations in the JGI *Z*. *tritici* database still need to be revised and that RNA-Seq analysis can significantly improve the published gene models. With the information on alternative gene models a significant insertion bias was detected, with intergenic (promoter and terminator) regions receiving more T-DNA hits than expected. In contrast, insertions into coding regions occurred less often, coinciding with results previously reported for large scale analyses of randomly-selected transformants in *M*. *oryzae* [52,53]. A similar pattern was detected in *Colletotrichum graminicola* [51]. As expected, all of the dimorphic switch deficient mutants obtained were drastically reduced or completely abolished in pathogenic development, since susceptible host plants inoculated with the mutants didn’t develop any symptoms associated with STB disease. Among the genes obtained, several were found to encode known pathogenicity factors, emphasizing the suitability of insertional mutagenesis in uncovering of pathogenicity factors. In this study, only when a T-DNA was inserted in an ORF or within 2.5 kb up- or downstream of an ORF, t was assumed that expression of the gene could be influenced by the T-DNA insertion and the gene was marked as a potential dimorphism-related (pathogenicity) gene. However, most of the genes encoding known virulence factors in *Z*. *tritici* were not identified in our screen pipeline, emphasizing the incompleteness of the transformant library. Functional categorization of the entire list of genes indicated that certain metabolic pathways, cell wall integrity, and certain cellular processes (protein translocation and degradation) appear to be important for dimorphism in *Z*. *tritici*. Furthermore, most of the genes were predicted to encode hypothetical proteins, pointing to novel avenues of dimorphic switch regulation, particularly specific for *Z*. *tritici*.

For instance, amongst the genes affected, one was predicted to encode a phospholipase A2. Generally, phospholipases A2 (PLA2s) belongs to a superfamily of enzymes catalyzing the hydrolysis of the sn-2 fatty acids of membrane phospholipids. These enzymes are suggested to exert multiple functions in maintaining the membrane phospholipid homeostasis and for production of a variety of lipid mediators, which in turn act as second-messenger in diverse cellular signalling events [54]. Comparison with publicly available data from other fungal species revealed a potential role for this gene in morphogenesis as well as in pathogenicity associated processes. For instance, in *Sporothrix shenkii* a link between G-protein mediated signalling and PLA2 has been evidenced. The heterotrimeric G-proteins seem to interact with the cytosolic phospholipase A2 (cPLA2), participating in the control of dimorphic switch [55]. Hence, using cPLA2 inhibitors, this enzyme was shown to affect stimulation of yeast-to-hyphal transition in *S*. *schenckii* by blocking re-entry into the yeast cell cycle. Two further genes potentially disrupted in *myco#5552* mutant included those putatively encoding Δ12-fatty acid desaturase (FAD) and methionine synthase respectively. Whether the observed inability of the mutant strain to undergo dimorphic switch is due to inactivation of the first gene or the second one, or rather the result of additive inactivation effect, remains speculative and requires further analysis. Nevertheless, the role of the homologous or related genes in several pathogenic fungi has been elucidated, suggesting that both of them can be involved in morphological processes associated with the hyphal formation. For instance, in the opportunistic pathogen *Candida parapsilosis* the role of Ole1 fatty acid desaturase (stearoyl-CoA desaturase), which synthesizes oleic acid, has been previously examined. Mutants disrupted in the gene exhibited diverse physiological aberrations including severely impaired pseudohyphal formation, drastically reduced virulence and hypersensitivity to macrophages and various stress-inducing factors, such as salts, SDS, and H_2_O_2_ [56]. Similarly, a partial repression of *OLE1* in *C*. *albicans* prevented hyphal development in aerobic conditions and blocked the formation of chlamydospores [57]. Methionine biosynthesis also appears crucial for dimorphism. For instance, methionine was previously shown to be implicated in promoting the dimorphic transition as it was observed that the presence of methionine induced the filamentous growth in *C*. *albicans* [58]. Likewise, in *Pichia fermentans*, methionine has been demonstrated, probably after its conversion to methanol, to enable the shift from yeast-like to pseudohyphal morphology *in vitro* [59].

Moreover, among the identified mutant strains through ATMT insertional mutagenesis, two were found to harbor T-DNA insertions in genes previously characterized and shown to be determinants of pathogenicity in different fungi. The flanking sequence from the *myco#1* mutant obtained was assigned to predicted *SSK1* homolog in *Z*. *tritici*. For the *myco#4* mutant the associated gene is predicted to encode Ade5,7 homolog previously characterized in *S*. *pombe*. Within this study we selected these two genes for functional characterization. Using targeted inactivation of the genes we proved the effect of the T-DNA insertion on the phenotype of the corresponding random mutants observed and validated the concept of our screening approach in identifying of dimorphism-related genes. Consistent with the presumptive implication of *ZtSSK1* in HOG-pathway we validated the involvement of the gene in the cellular responses upon osmotic and oxidative stress. Similarly we could confirm the responsibility of the Ade5,7 homologous gene in *Z*. *tritici* for the auxotrophic property of the mutant.

### *ZtSSK1* is involved in oxidative and osmotic stress responses and responsible for fludioxonile sensitivity

Thus, targeted inactivation of *ZtSSK1* in *Z*. *tritici* resulted in a ROS-sensitive phenotype, similar to that observed in other fungal organisms previously reported [60,61,44]. Apart from ROS resistance, an important role for a *ZtSSK1* response regulator in cellular tolerance to osmotic stress also was demonstrated in the present study. Thus, *Z*. *tritici* mutants impaired for *ZtSSK1* display increased hypersensitivity to NaCl and sorbitol, a phenotype highly resembling the mutants lacking *ZtHOG1* [21]. The involvement of *ZtSSK1* in osmotic stress resistance is supported further by the fact that the Δ*Ztssk1* mutants produced very few or no protoplasts after incubating with cell wall-degrading enzymes. This stays in agreement with results previously reported by Mehrabi *et al*., demonstrating that *ZtHOG1* deficient mutant strains of *Z*. *tritici* also failed to generate viable protoplasts. Generally, the finding that Δ*Ztssk1* mutants are impaired in their ability to cope with environmental stress is consistent with the prediction that *ZtSSK1* is involved in stress response and regulation. In addition we could also confirm the previously reported requirement of HOG-pathway for fungal development and in particular for cell wall / plasma membrane biogenesis. We stated an enhanced lysis rate of conidia collected from Δ*Zthog1* and Δ*Ztssk1* mutant strains when treated with CWDE. This was also supported by *in vitro* growth test on SDS medium, showing elevated sensitivity of the mutant strains against this membrane perturbing agent compared to wildtype. Moreover, as previously shown for Δ*Zthog1*, the inactivation of *ZtSSK1* also resulted in resistance to fludioxonil fungicide, indicating the interaction among the signaling components in terms of fungicide sensitivity. These results also stay in concordance with those previously obtained for *Alternaria alternata*, showing that *AaSSK1* is involved in ROS resistance, osmotic resistance, fungicide sensitivity and fungal virulence [44]. As expected, *ZtSSK1* was also found to play an important role in the establishment of plant colonization because gene inactivation mutants were reduced in their ability to induce necrotic lesions on susceptible wheat cultivar Riband. The explanation for this observation is due to the impaired ability of Δ*Ztssk1* mutants to undergo dimorphic transition leading to decreased colonization of host tissue. In addition, the elevated sensitivity to ROS observed may be causative for this phenotype.

### Inactivation of *MYCO4* results in purine auxotrophy

An interesting phenotype was observed in case of the *myco#4* random mutant. The gene in which the corresponding mutant was disrupted was found to encode a bifunctional phosphoribosylglycinamide synthetase (glycinamide ribotide synthase/aminoimidazole ribotide synthase; indicated as GART*), which is known for the second and fifth catalyzing step of *de novo* purine biosynthesis in *Schizosaccharomyces pombe* [62]. *De novo* purine biosynthesis has been extensively studied in different organisms, including fungi, bacteria, plants and animals, particularly in much detail in *Escherichia coli* and baker’s yeast *S*. *cerevisiae* and has been reported to be highly conserved in evolution, even between prokaryotes and eukaryotes [63–65].

Since our preliminary BLAST analysis indicated a high degree of conservation of purine biosynthetic enzymes in *Z*. *tritici* with those of distantly related *Schizosaccharomyces pombe* as well as closely related filamentous fungi such as *Neurospora crassa*, *Magnaporthe oryzae*, *Aspergillus nidulans* and *Fusarium graminearum*, it is highly conceivable that a similar pathway is present in *Z*. *tritici*.

**Fig 10:**
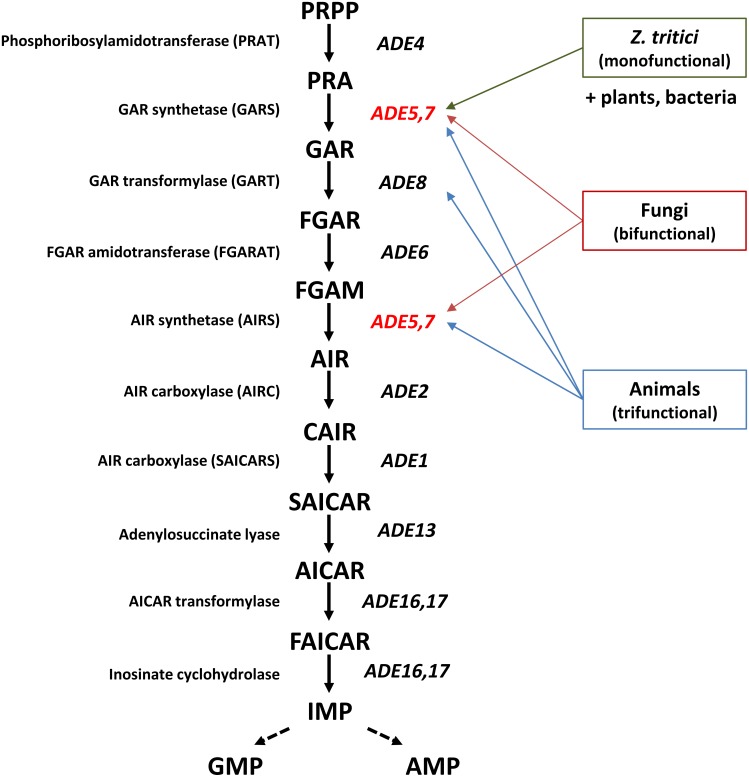
Schematic representation of the *de novo* purine biosynthesis in the yeast *Saccharomyces cerevisiae*. Abbreviations: PRPP: 5-phospho-ribosyl-1α-pyrophosphate, PRA: 5-phospho-β-D-ribosylamine, GAR: 5-phosphoribosylglycinamide, FGAR: 5'-phosphoribosyl-N-formyl glycinamide, FGAM: 5'-phosphoribosyl-N-formylglycinamidine, AIR: 5'-phosphoribosyl-5-aminoimidazole, CAIR: 5'-phosphoribosyl-5-aminoimidazole-4-carboxylate, SAICAR: 5'-phosphoribosyl-4-(N-succinocarboxamide)-5-aminoimidazole, AICAR: 5'-phosphoribosyl-4-carboxamide-5-aminoimidazole, FAICAR: 5'-phosphoribosyl-4-carboxamide-5-formamidoimidazole, IMP: inosine monophosphate, AMP: adenosine monophosphate, GMP: guanosine monophosphate. The *S*. *cerevisiae* gene *ADE5*,*7* (depicted in red) is homologous to *Z*. *tritici MYCO4*. In contrast to bifunctional yeast counterpart, the *Z*. *tritici* has only GARS activity (monofunctional). The animal counterpart is predicted to encode a bifunctional enzyme complex, exhibiting GARS, AIRS and FGAR activities. The trifunctional human GART* is highlighted by blue box.

Interestingly, the structural analysis of the deduced protein sequences revealed a diverged structure (and function) of the corresponding protein orthologs. The evidence for this is provided by comparison of deduced Myco4p with bifunctional homologs of other fungi including *S*. *cerevisiae* and the trifunctional mammalian homolog. Hence, InterProScan analysis revealed for *Z*. *tritici* and closely related fungi from *Mycosphaerellaceae* family only GARS domains responsible for conversion of 5-phospho-β-D-ribosylamine (PRA) to 5-phosphoribosylglycinamide (GAR), in contrast to other fungal representatives harbouring an additional AIRS domain (Fig B in S1 File). Meanwhile, the *Homo sapiens* homolog combines the functions of Ade5,7p, catalyzing the second and fifth steps of the *de novo* purine biosynthesis in analogy to fungal representatives, but owns additional domain responsible for the conversion of 5-phosphoribosylglycinamide (GAR) to 5-phosphoribosyl-N-formylglycinamide (FGAR). This finding is intriguing since the monofunctional members are only common for bacteria and plants. Hence, the three steps mentioned are catalyzed by three enzymes in bacteria and plants, but two in fungi (Fig 10). The reason for the predominant monofunctional variant of the GART* protein in *Zymoseptoria* clade is currently unknown and the biological role for this phenomenon remains to be elucidated. In light of the assumed structural difference, but at the same time retained functional conservation of the enzyme, the question remains, however, whether it is possible to identify inhibitors that are specific to the fungal GART*s.

However, this structural/functional difference observed may result in differentially coordinated regulation of the three enzymatic activities (assigned to three different enzymes in *Z*. *tritici*) with global impact on metabolic processes. The remaining two genes encoding AIRS (Protein ID: 74864) and GART (Protein ID: 73624) were found in *Z*. *tritici* genome.

The striking difference in conformational structure suggested between mammalian and fungal orthologs implies that Myco4p may be regarded as a potential therapeutic target for the development of novel antifungal drugs. In fact, because the human GART requires 10-formyl-tetrahydrofolate (THF) as a cofactor, the corresponding part of the trifunctional enzyme has been addressed as an anticancer target for THF analogues by inhibiting its activity [66]. This difference emphasizes the phylogenetic divergence of the fungal GART*s and their human counterparts. As expected, the targeted inactivation of the gene in *Z*. *tritici* resulted in auxotrophic phenotype of the generated mutant strains. Without supplemented adenine or hypoxanthine none of the targeted mutant strains were able to grow on N-deprivation basal medium, lacking the external source of purines. However when providing external purines, even at low concentration the wildtype growth could be restored, indicating that the salvage purine pathway was operational in the mutants. Collectively, our observations lead to the conclusion that the impaired purine (adenine) biosynthesis in *MYCO4* deficient mutant strain affects cellular functions associated with growth and development, and appears to be causative for abolished pathogenicity. Previously, the role of *de novo* purine biosynthesis has also been addressed in dimorphic systemic fungi like *C*. *albicans* and *C*. *neoformans*, showing that disruption of genes involved in this pathway results in attenuated or complete loss of virulence in mammalian models and general growth defects [67,68]. Consequently, this implies that Myco4p represents a virulence factor necessary for disease establishment. Questionable is, however, the ability of mutant strain to use the exogenous purine nucleotides *in planta*, since our results indicate that growth of the mutant strain is restored by supplementing the synthetic media with exogenous adenine/hypoxanthine sources even at lower concentrations compared to those that are present in the plant apoplast. Therefore, further investigations should be carried out in order to analyze the ability/inability for the exogenous purine uptake *in planta*.

Additionally, the attributed traits concerning the auxotrophy of the mutant strain open up a further possibility which could be advantageous for the utilization within biotechnological and molecular biological applications. Complementation of the *MYCO4*-disruption strain with the intact copies of *MYCO4* suggests that there could be a potential use of this gene as an auxotrophic selectable marker for genetic transformation in *Z*. *tritici*, as was previously shown for *Giberella* zeae (*Fusarium graminearum*) [69]. In general, genes responsible for adenine or arginine auxotrophic phenotype in other fungi have been broadly used as selection markers for transformation systems in *Pichia pastoris*, *Aspergillus oryzae* and *Saccharomyces cerevisiae* [70,71]. The usage of *MYCO4* in a similar way would undoubtedly extend the arsenal of selection markers used today for transformation and functional genomics of *Z*. *tritici*.

## Conclusion

In conclusion, T-DNA insertional mutagenesis has been validated as a means of identifying individual genes regulating the ability of *Z*. *tritici* to perform the dimorphic switch and to cause disease. By providing a list with potential candidate genes (Table 1), we emphasized some dimorphism related processes which should receive a broader attention for further investigation. Undoubtedly, these data provide a solid basis for accelerating research on this economically important phytopathogenic fungus *Z*. *tritici* and generally on the process underlying the dimorphic transition, which also has a huge impact on pathogenic development of systemic dimorphic fungi presenting a serious global health threat for humans. Verification and characterization of further potential pathogenicity genes obtained is in progress. Together, these efforts should lead to a comprehensive picture of the molecular requirements for dimorphism in *Z*. *tritici*. The identification of *ZtSSK1* and *MYCO4 (ADE5*,*7)*, which have previously been shown to have functions associated with pathogenicity, validates the procedure set out in the study.

We confirmed the canonical role of *SSK1* homolog in *Z*. *tritici*, being constituent of HOG-pathway, concerning its implication in the osmotic/oxidative stress response and involvement in cell wall / plasma membrane biogenesis. The impaired purine (adenine) biosynthesis in *MYCO4* deficient mutant strain affects cellular functions associated with growth and development, and appears to be causative for abolished pathogenicity. Consequently, this implies that both gene products represent virulence factors necessary for dimorphic transition and disease establishment. The outcome from our results provides an excellent basis for further extended investigations to deepen our understanding of molecular mechanisms associated with dimorphism.

## Supporting information

S1 File This file contains a summary of primers used in this study, information on cloning strategy for generation of gene inactivation constructs and Southern blot analysis as well as results of phenotypic assays. (DOCX)
