# Supplementary material for: Identification of factors involved in dimorphism and pathogenicity of Zymoseptoria tritici
Source: PLoS One. 2017 Aug 22;12(8):e0183065. doi: 10.1371/journal.pone.0183065 (PMC5568738; doi:10.1371/journal.pone.0183065)
Supplement: S1 File — (DOCX) [file pone.0183065.s001.docx]

# Supporting information

### Summary of primers used in this work

**Table A: Primers used for generation of gene inactivation constructs.** Lower case sequences represent the tails of the primers used for overlapping of fragments within Gibson assembly reaction.

| **Primer name** | **Direction** | **Sequence (5‘ to 3‘)** |
| --- | --- | --- |
| **myco1-fr.1-for** | sense | gaattaattcctaggccaccatgttgggccTGACGAAATGATTGCCAGTTATATTTTTC |
| **myco1-fr.1-rev** | antisense | ttcaatatcatcttcGGAGAACTCGGATCAAGGCAAG |
| **myco1-fr.2-for** | sense | tgatccgagttctccGAAGATGATATTGAAGGAGCATTTTTG |
| **myco1-fr.2-rev** | antisense | tctcctccagctcgcTCTTGTTCGGTCGGCATCTAC |
| **myco1-fr.3-for** | sense | gccgaccgaacaagaGCGAGCTGGAGGAGAAGCAG |
| **myco1-fr.3-rev** | antisense | acgtggtggtggtggtggtggctagcgttaacaGGACAGCAGAGAGGTCCTTCTC |
| **myco4-5‘-for** | sense | CCATTCGATGCGACGCTGGC |
| **myco4-5‘-rev** | antisense | TTGCCGACGAGGAGGATGCG |
| **myco4-3‘-for** | sense | CTTCTCATACCTGGCCCGGAAGC |
| **myco4-3‘-rev** | antisense | AGGATTGAGTCTCCGGGTCGCC |

**Table B: Primers used for genome walking analysis**

| Primer name | Direction | Sequence (5‘ to 3‘) |
| --- | --- | --- |
| gspAa | antisense | TCGCGGTGAGTTCAGGCTTTTTCATGATCG |
| gspA | antisense | CGCACAAGTTATCGTGCACCAAGCAGCAGA |
| gspBa | sense | TCAGAGCTTGGTTGACGGCAATTTCGATGA |
| gspB | sense | ATGGCTGTGTAGAAGTACTCGCCGATAGTG |
| PPR1 | - | **PO_4_**-CTAGGGCCACCACG-**NH_2_** |
| Pad1 | - | GTAATACGACTCACTATAGGGCACGCTGGTGGCC |
| PP1 | - | GTAATACGACTCACTATAGGGC |
| PPR2 | - | **PO_4_**-CCGGTGCCACCACG-**NH_2_** |
| Pad2 | - | GTAATACGACTCACTATAGGGCACGCGTGGTGGCA |
| PP2 | - | ACTATAGGGCACGCGTGGT |

**Table C: Primers used for complementation constructs**

| Primer name | Direction | Sequence (5‘ 🡪 3‘) |
| --- | --- | --- |
| myco1-Comp-for | sense | AAGCCGCCGGTCCCTTAGGT |
| myco1-Comp-rev | antisense | GCTCGAAGGCGAGTGGCAGG |
| myco4-Comp-for | sense | CGCTCATTGCGAACAGCAGGC |
| myco4-Comp-rev | antisense | TCGGCGACCACTTCGGTGTC |

**Table D: Primers used for probe generation (for Southern Blot analysis)**

| Primer name | Direction | Sequence (5‘ 🡪 3‘) |
| --- | --- | --- |
| myco1-probe-for | sense | TCGTGAGGCATGGATGAAGT |
| myco1-probe-rev | antisense | CTAACGAACCAAGCGAGCAG |
| myco4-probe-for | sense | ACGGCACAACATCCCGACTGC |
| myco4-probe-rev | antisense | GCTACTGGTGCCGGTGCGTA |
| HPT-probe-for | sense | CGAAGAATCTCGTGCTTTCAGC |
| HPT-probe-rev | antisense | CCAGAAGAAGATGTTGGCGAC |

**Table E: Primers used for Screen PCR**

| **Primer name** | **Direction** | **Sequence (5‘ to 3‘)** |
| --- | --- | --- |
| **myco1-Comp-for** | sense | AAGCCGCCGGTCCCTTAGGT |
| **myco1-Comp-rev** | antisense | GCTCGAAGGCGAGTGGCAGG |
| **myco4-5‘-for** | sense | CCATTCGATGCGACGCTGGC |
| **myco4-3‘-rev** | antisense | AGGATTGAGTCTCCGGGTCGCC |


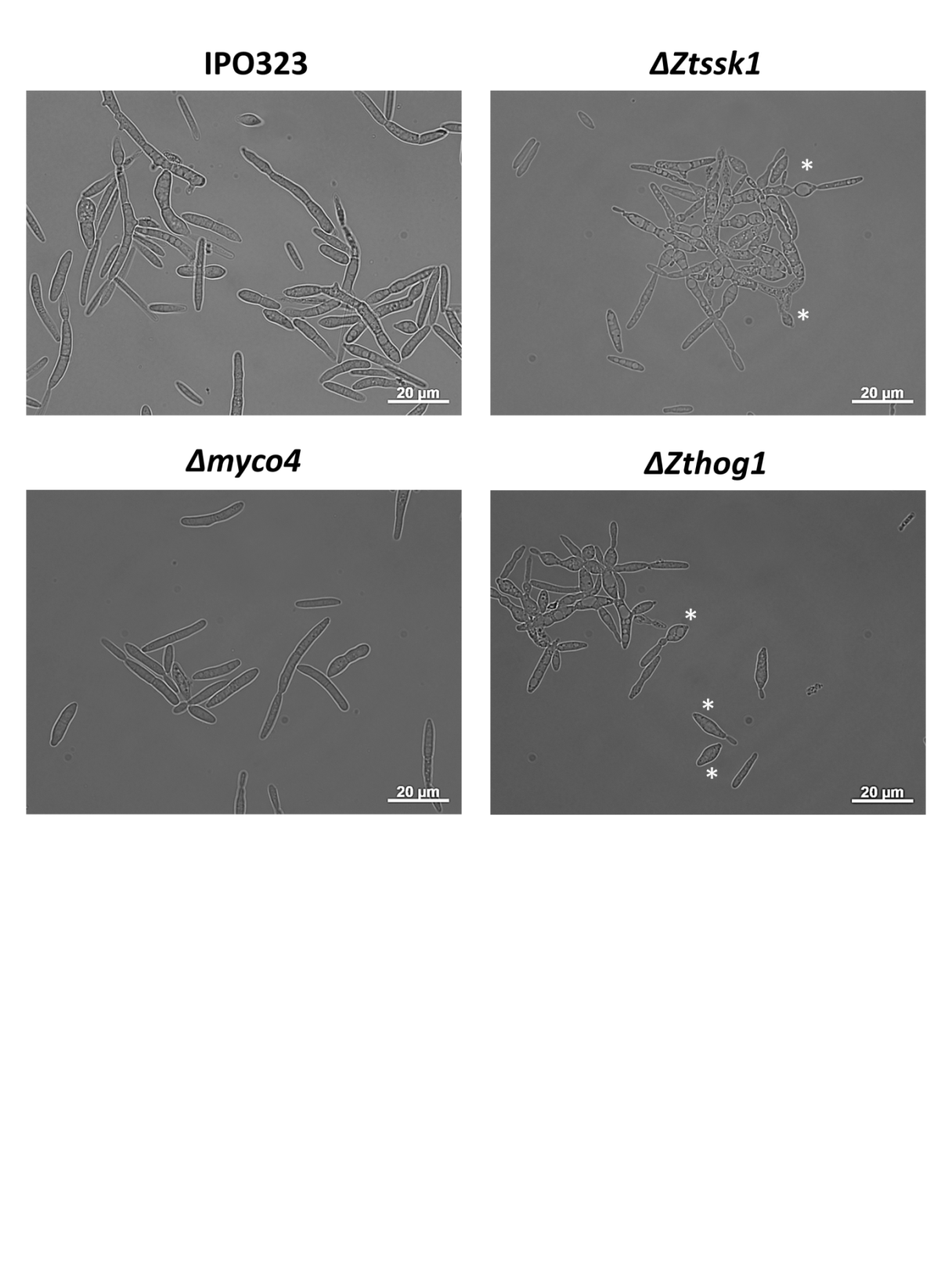


**A**

**B**

**
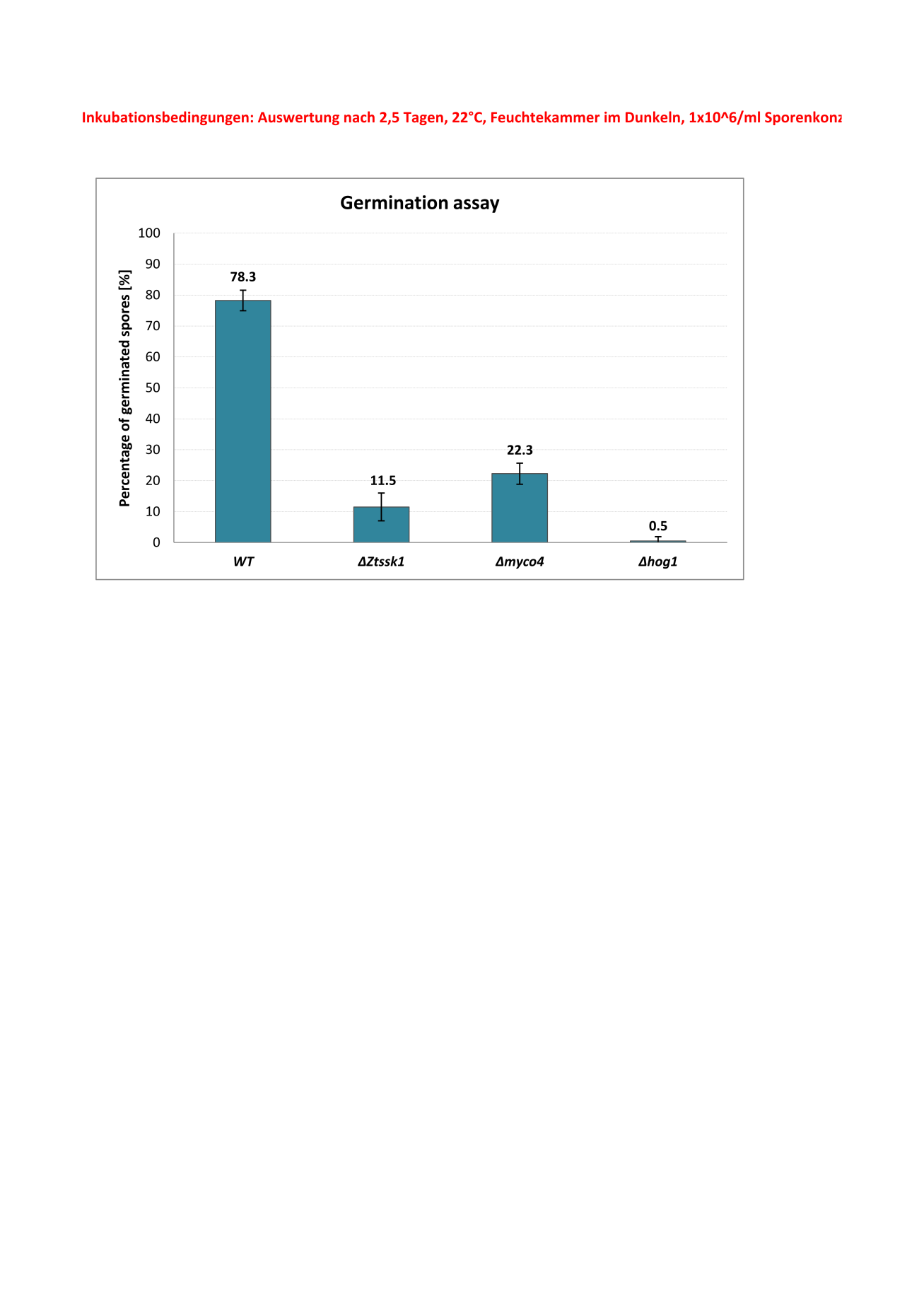
**

**Fig A:** **(A)** **Comparative phenotyping of *Zymoseptoria tritici* mutants on microscopic scale.** The strains were previously icubated in YEG at 18 °C under daylight settings for 3 days. The *∆myco4* disruptants produce wildtype-like spores in YEG. White asterisks depict irregular shaped, ovoid conidia in case of *∆Zthog1* and *∆Ztssk1*, consistent with phenotypes previously reported for homologous mutant strains in other fungi. **(B)** ***In vitro* germination assay of wildtype strain IPO323 (WT) and targeted mutant strains.** For each strain 100 µl of spore suspension at final concentration of 10^6^/ml were spotted on microscopic slide covered with a thin layer of H_2_O agar and incubated in the dark chamber with nearly full-saturated humidity at 18 °C. The ratio of germination was calculated as the mean percentage of conidia germinated after 72 h. For each strain three biological replicates were analyzed.

*
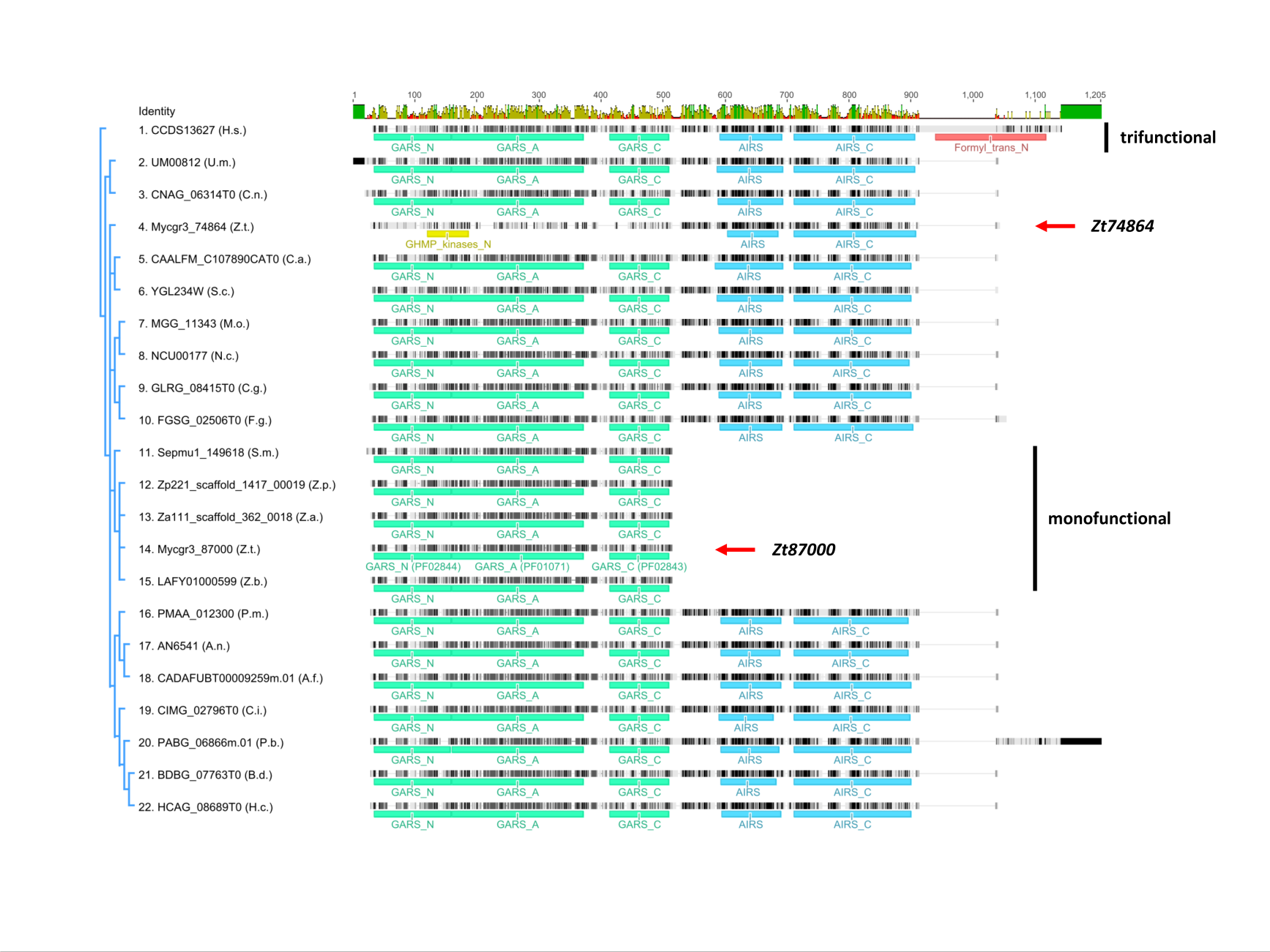
*

**Fig B:** **Protein sequence analysis of deduced Myco4p homologs.** Multiple sequence alignment analysis of deduced protein sequences was performed by MUSCLE with default parameters. Identical amino acids are shaded in black, conserved amino acids in dark gray and similar amino acids in light gray. The adjacent phylogenetic tree was constructed using “neighbour-joining”-algorithm based on the “Jones-Taylor-Thornton (JTT)” - model. The statistical accuracy was tested by bootstrap analysis including 100000 replicates. Protein sequences derived from the [*Mycosphaerellaceae*](https://en.wikipedia.org/wiki/Mycosphaerellaceae) family are clustered in one clade since they harbour only *GARS* functional domain (comprised of *GARS_N*, *GARS_A* and *GARS_C*) predicted by InterProScan. All other fungal representatives including yeast possess bifunctional proteins, indicated by the presence of two additional functional domains *AIRS* and *AIRS_C* (depicted in blue). Red arrows indicate Myco4p (*Zt87000*) and one predicted protein (*Zt74864*) with *AIRS* domain. Both appear monofunctional, but may together reconstitute the bifunctionality of yeast counterpart The human ortholog is trifunctional due to the presence of *Formyl_trans_N* domain (highlighted in red), additionally conferring the GART activity. Orthologs from the following organisms were used: *Aspergillus nidulans* FGSC A4 (*A.n.*), *Aspergillus fumigatus* A1163 *(A.f.)*, *Blastomyces dermatitidis* SLH14081 *(B.d.)*, *Candida albicans* WO-1 (*C.a.*), *Coccidioides immitis* RS (*C.i.*)*, Colletotrichum graminicola* M1.001 (*C.g.*), *Fusarium graminearum* PH-1 (*F.g.*), *Histoplasma capsulatum* NAm1 *(H.c.*), *Magnaporthe oryzae* 70-15 (*M.o.*), *Neurospora crassa* OR74A (*N.c.*), *Paracoccidioides brasiliensis* Pb03 (*P.b.*), *Sphaerulina musiva* SO2202 (*S.m.*), *Talaromyces marneffei* ATCC 18224 (*P.m.*), *Ustilago maydis* (*U.m.*), *Zymoseptoria ardabiliae* STIR04_1.1.1 *(Z.a.*), *Zymoseptoria brevis* ZBREZB163 *(Z.b.*) and *Zymoseptoria pseudotritici* STIR04_2.2.1 (*Z.p.*). *Saccharomyces cerevisiae* (*S.c.*) and *Homo sapiens* (*H.s.*) were used as outgroup.

***ZtSSK1* – Strategy used for generation of the *∆Ztssk1* using targeted gene inactivation and**

**Southern Blot providing evidence of gene replacement**


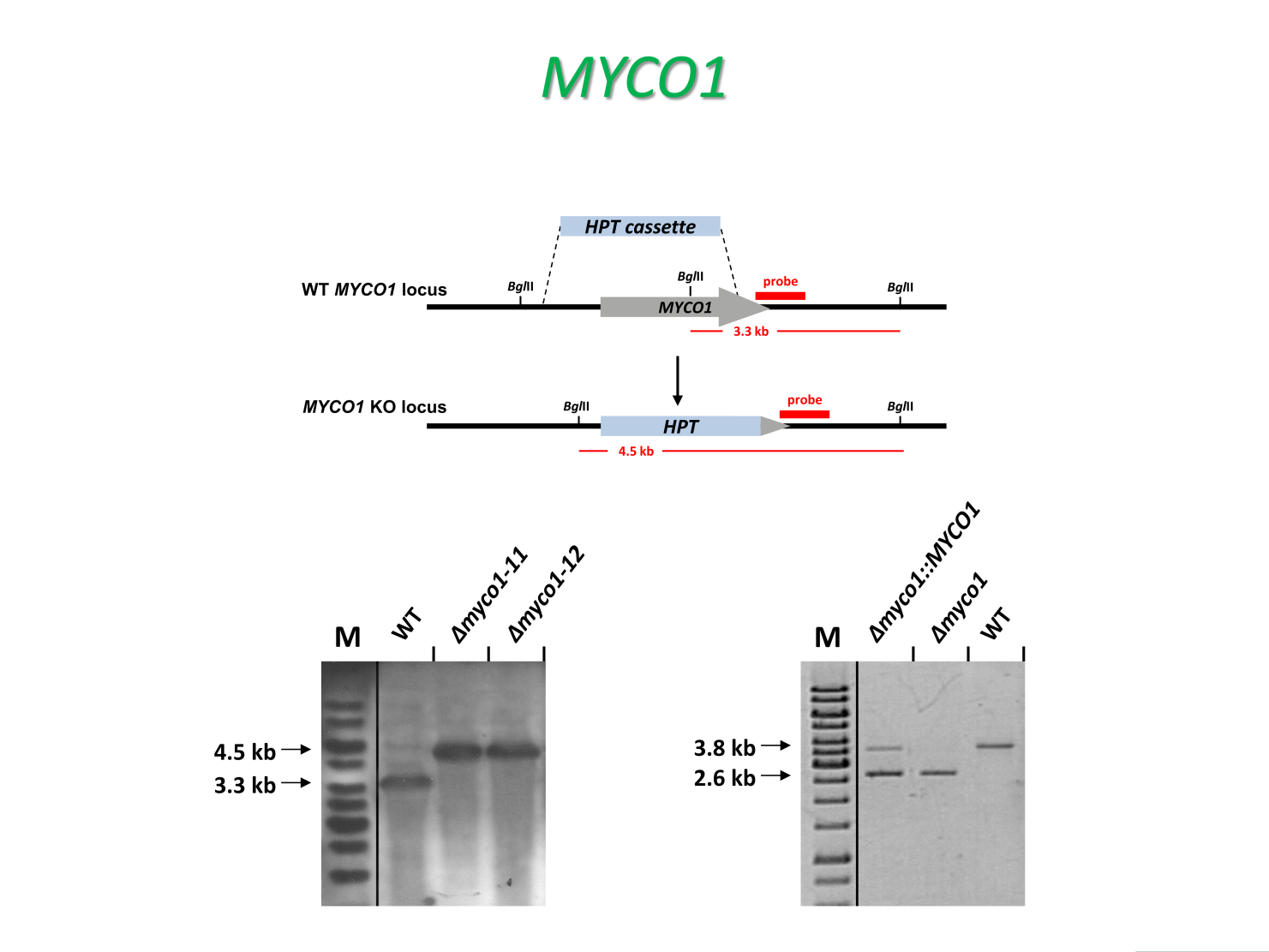


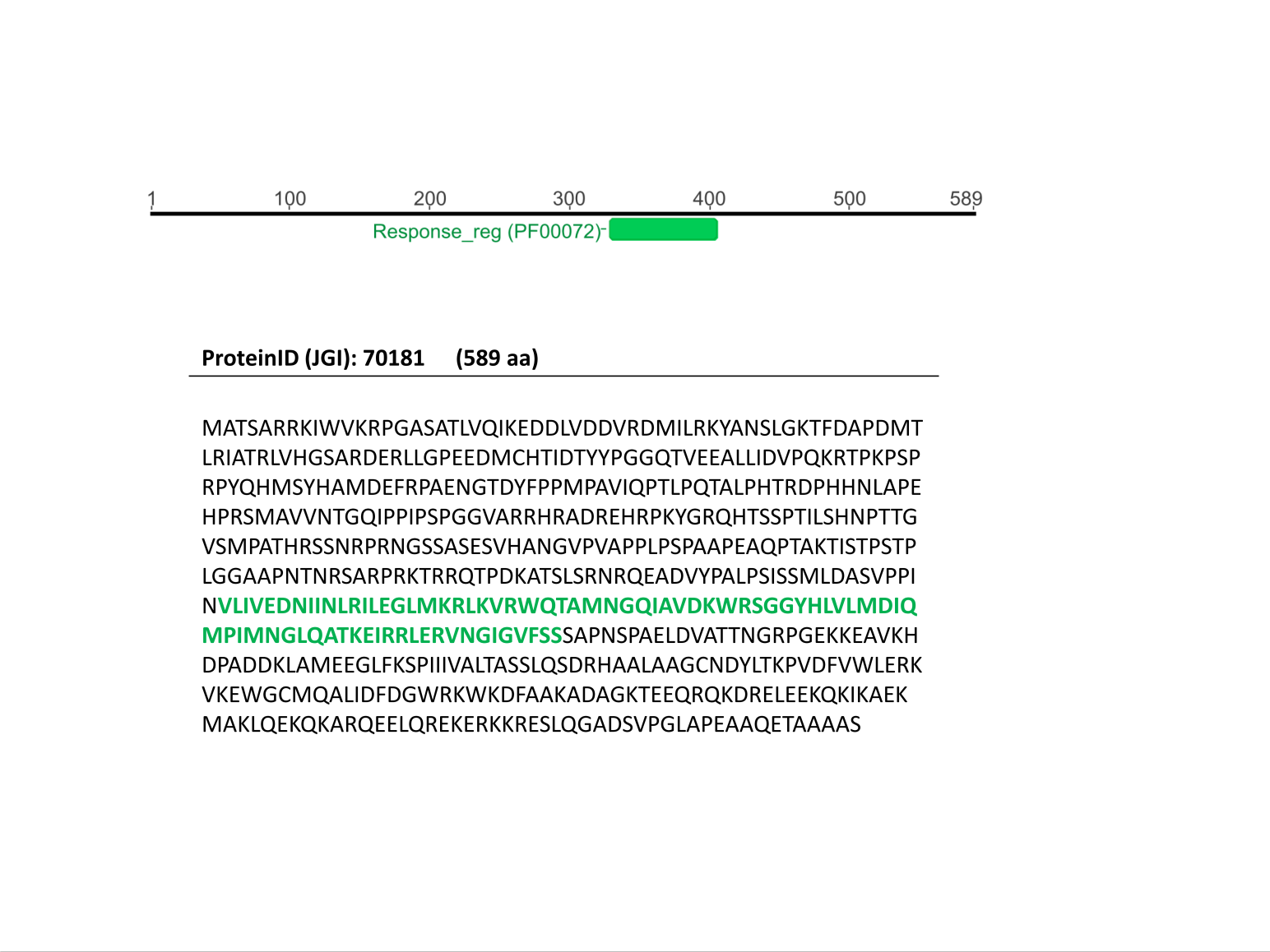


**Fig C:** **Targeted gene inactivation of *ZtSSK1***. **(A)** Gene structure of ***ZtSSK1* (*=MYCO1*)** with functional domain predicted by InterProScan as well as adjacent protein sequence deduced are shown. Sequence region matching the predicted domain is highlighted in green. **(B)** Construction of the gene inactivation construct and targeted gene disruption by replacing a significant portion of ORF of the gene with *HPT* cassette. Location of genomic sequence for probe hybridization and restriction sites used for Southern Blot are indicated. **(C)** Southern Blot analysis. Genomic DNA derived from wildtype strain IPO323 and potential *ΔZtssk1* mutants was restricted with *Bgl*II and then probed with a 300 bp fragment amplified with the primers . **(D)** Screen-PCR was employed to confirm the loss of functional copy of *ZtSSK1* gene in the *ΔZtssk1* mutant and the presence of additional gene copy in the complementation strain *ΔZtssk1*/*ZtSSK1*, using the primers myco1-fr.1-for and myco1-fr.3-rev.

***MYCO4* – Strategy used for generation of the *∆myco4* using targeted gene inactivation and**

**Southern Blot providing evidence of gene replacement**


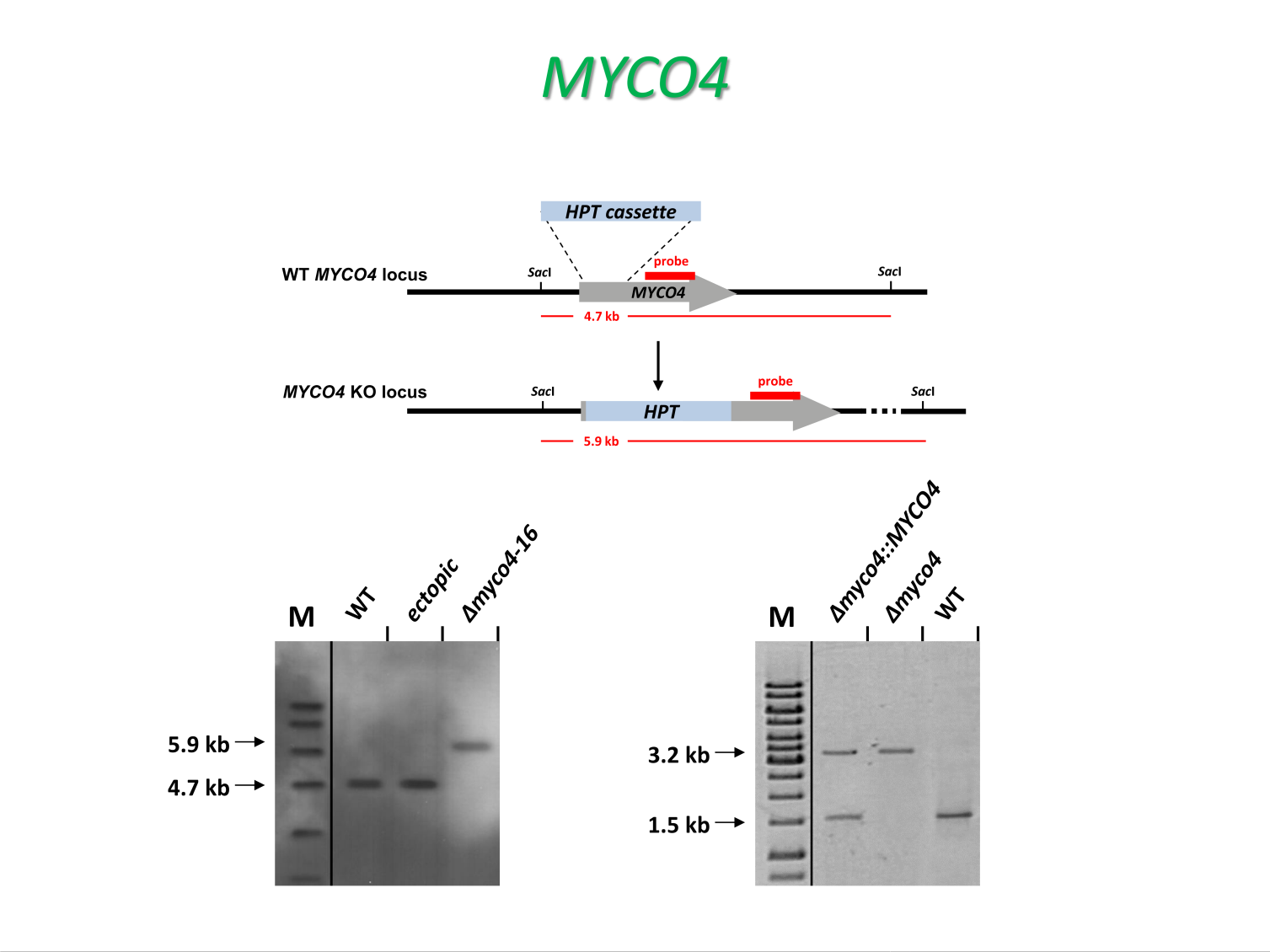


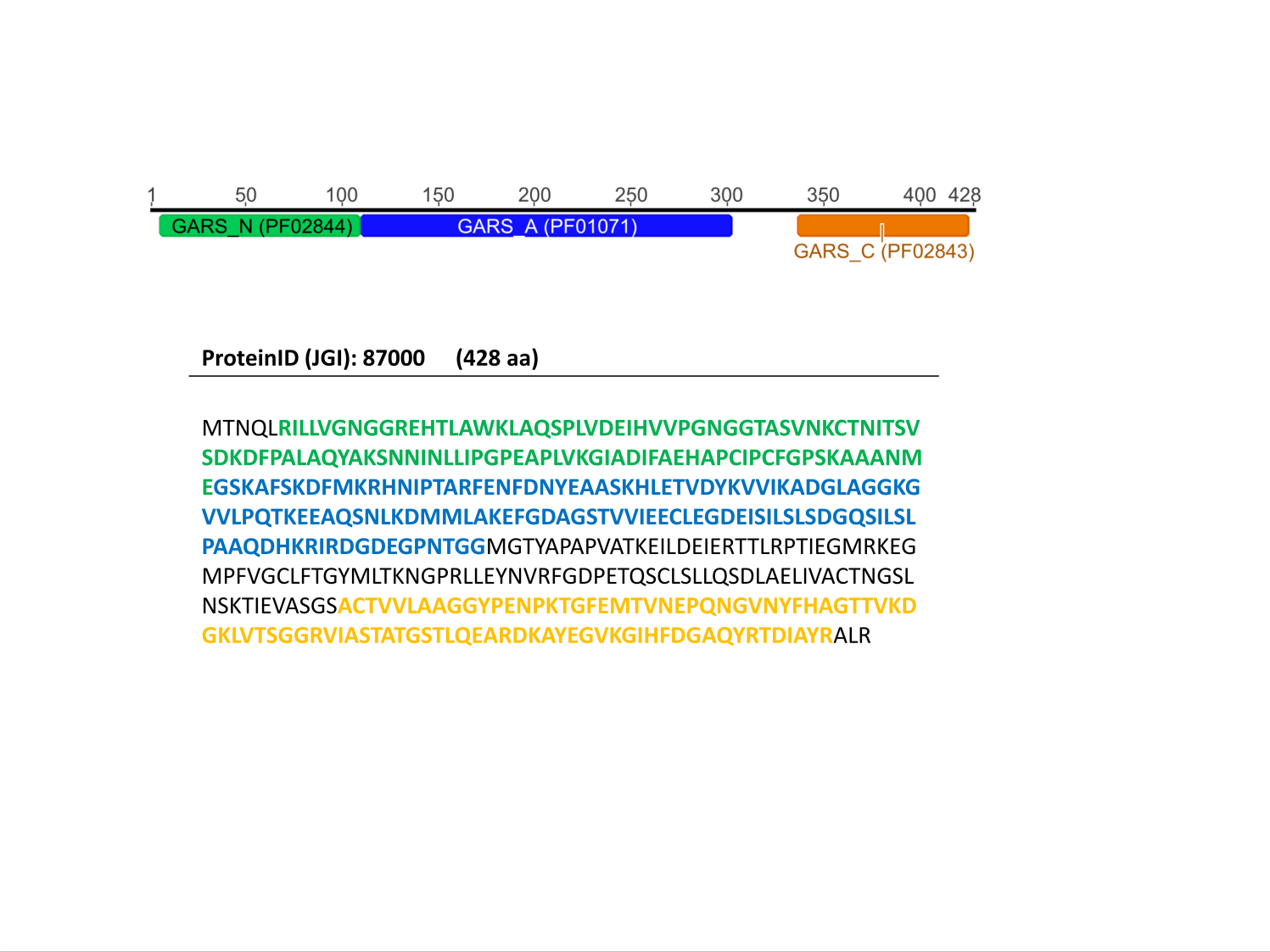


**Fig D:** **Targeted gene inactivation of *MYCO4***. **(A)** Gene structure of *MYCO4* with functional domain predicted by InterProScan as well as adjacent protein sequence deduced are shown. Sequence regions matching the predicted domains are highlighted in respective colours. **(B)** Schematic representation of targeted gene disruption by replacing a significant portion of ORF of the gene with *HPT* cassette. Location of genomic sequence for probe hybridization and restriction sites used for Southern Blot are indicated. **(C)** Southern Blot analysis. Genomic DNA derived from wildtype strain IPO323 and potential *Δmyco4* mutants was restricted with *Sac*I and then probed with a 378 bp fragment amplified with the primers myco4-probe-for and myco4-probe-rev. **(D)** Screen-PCR was employed to confirm the loss of functional copy of *MYCO4* gene in the *Δmyco4* mutant and the presence of additional gene copy in the complementation strain *Δmyco4*/*MYCO4*, using the primers myco4-5‘-for and myco4-3‘-rev.

### Plasmid construction for targeted inactivation and complementation of the target genes in *Zymoseptoria tritici*

*ZtSSK1* (*=MYCO1*) (JGI Protein ID: 70181):

The construction of transformation vector used for inactivation of *MYCO1* gene was employed using the Gibson Assembly® approach. To this end, three required fragments were amplified by PCR using the primer pairs containing the overhangs overlapping with the neighboring fragments. The primer pairs myco1-fr.1-for and myco1-fr.1-rev as well as myco1-fr.3-for and myco1-fr.3-rev were used to amplify fragments representing the ORF sequence regions of the *MYCO1* gene suitable for homologous recombination using genomic DNA as template. For the amplification of *HPT* cassette from the pCB1636 the primers myco1-fr.2-for and myco1-neu-fr.2-rev were used. Thereby it was possible to generate the desired final gene inactivation vector pCAMB-myco1-HPT-Final within a single assembly reaction. The generated transformation plasmid was verified by restriction analysis and could be directly used for ATMT.

For complementation of the *MYCO1***-**inactivated mutant the construct pCAMB-myco1-Comp-BAR was generated by amplification of the WT gene locus from strain IPO323 using the primers myco1-Comp-for and myco1-Comp-rev. The amplified PCR product was ligated to pGEM-T easy to give pGEM-myco1-Comp. Subsequently the insert excised using *Not*I was cloned into the vector pCAMB-BAR (7747 bp) restricted with *Psp*OMI. The generated final vector pCAMB-myco1-Comp-BAR was verified by restriction analysis and could be directly used for transformation of hygromycin resistant mutants.

*MYCO4* (JGI Protein ID: 87000):

For inactivation of *MYCO4* gene the primers myco4-5’-for, myco4-5’-rev and myco4-3’-for, myco4-3‘-rev were used to generate two PCR products, representing the gene flank regions with 616 bp and 700 bp respectively. The 5’-flank was subsequently subcloned into the vector pJet to generate pJet-myco4-5’-correct (3590 bp). The 3’-fragment (700 bp) was ligated into pGEM-T easy giving the resulting vector pGEM-myco4-3’ (3717 bp).

pGEM-myco4-3‘ was restricted with the enzyme combination *EcoRI+Spe*I and a 700 bp fragment was cloned into *EcoRI+Avr*II restricted pCAMB-HPT(*Sal*I) giving the resulting vector pCAMB-myco4-3‘-HPT. Finally the *Bgl*II*+Xba*I restricted fragment of pJet-myco4-5‘-correct was cloned into *Bgl*II*+Spe*I resctricted vector pCAMB-myco4-3‘-HPT to give the completed gene inactivation vector pCAMB-myco4-HPT-Final. The generated transformation plasmid was verified by restriction analysis and could be directly used for ATMT.

For complementation of the *MYCO4-*inactivated mutant the construct pCAMB-myco4-Comp-BAR was generated by amplification of the WT gene locus from strain IPO323 using the primers myco4-Comp-for and myco4-Comp-rev. The amplified PCR product was ligated to pGEM-T easy. The resulting pGEM-myco4-Comp vector was restricted by using *Not*I and a 4394 bp fragment was cloned into pCAMB-BAR (7747 bp) restricted with *Psp*OMI. The generated final vector was verified by restriction analysis and could be directly used for transformation of hygromycin resistant mutants.
